# “Friending” Teens: Systematic Review of Social Media in Adolescent and Young Adult Health Care

**DOI:** 10.2196/jmir.3692

**Published:** 2015-01-05

**Authors:** Lael M Yonker, Shiyi Zan, Christina V Scirica, Kamal Jethwani, T Bernard Kinane

**Affiliations:** ^1^Massachusetts General HospitalDepartment of PediatricsBoston, MAUnited States; ^2^Harvard Medical SchoolBoston, MAUnited States; ^3^Partners HealthcareCenter for Connected HealthBoston, MAUnited States

**Keywords:** social media, social networking sites, adolescents, young adults, health

## Abstract

**Background:**

Social media has emerged as a potentially powerful medium for communication with adolescents and young adults around their health choices.

**Objective:**

The goal of this systematic review is to identify research on the use of social media for interacting with adolescents and young adults in order to achieve positive health outcomes.

**Methods:**

A MEDLINE/PubMed electronic database search was performed between January 1, 2002 and October 1, 2013, using terms to identify peer-reviewed research in which social media and other Web 2.0 technologies were an important feature. We used a systematic approach to retrieve papers and extract relevant data.

**Results:**

We identified 288 studies involving social media, of which 87 met criteria for inclusion; 75 studies were purely observational and 12 were interventional. The ways in which social media was leveraged by these studies included (1) observing adolescent and young adult behavior (n=77), (2) providing health information (n=13), (3) engaging the adolescent and young adult community (n=17), and (4) recruiting research participants (n=23). Common health topics addressed included high-risk sexual behaviors (n=23), alcohol, tobacco, and other drug use (n=19), Internet safety (n=8), mental health issues (n=18), medical conditions (n=11), or other specified issues (n=12). Several studies used more than one social media platform and addressed more than one health-related topic.

**Conclusions:**

Social media technologies offer an exciting new means for engaging and communicating with adolescents and young adults; it has been successfully used to engage this age group, identify behaviors, and provide appropriate intervention and education. Nevertheless, the majority of studies to date have been preliminary and limited in their methodologies, and mostly center around evaluating how adolescents and young adults use social media and the resulting implications on their health. Although these explorations are essential, further exploration and development of these strategies into building effective interventions is necessary.

## Introduction

Health care providers (HCPs) face several important challenges in caring for the adolescent and young adult population. Developmentally, adolescents and young adults are in the midst of a stage in life during which they are striving to establish a sense of independence and self-identity, while also aiming to “fit in” and gain acceptance from their peers [1]. It is a critical time when health-risk behaviors (eg, substance use and high-risk sexual behaviors) are often initiated [2,3]. It is also a time of life transitions, such as moving away from parental control and establishing independent relationships with HCPs. Adolescents and young adults have access to more health information than in the past and possess the capacity to take an active role in tasks such as self-monitoring their health and adhering to medications [4]. However, the interplay between developmental factors and the overall transience of this population can contribute to a lack of communication between these young individuals and their HCPs; young people may want to discuss issues around their health with HCPs, but often do not [5].

On the other hand, adolescents and young adults are the most well-represented population online, with over 95% accessing the Internet daily [6,7]. Young people are also the earliest adopters and heaviest users of the newest Internet communication technologies such as social media, which in recent years has become increasingly accessible as a result of the widespread adoption of mobile and wireless Internet access. In fact, 81% report that they use social media and 67% report using it at least once a day [8]. Furthermore, as active social media users, 88% report sending instant messages, 87% have commented on a friend’s post, 86% have posted a status update, and 80% have posted a photo or video online [9]. Social media, by its nature of open sharing, collaboration, and exchange of user-generated content, has been shown to be useful in the creation and maintenance of social networks that are important in the spread of health behaviors [10-12]. Thus, to positively impact the care of young people, HCPs first need to recognize and understand the virtual landscape where they reside to ensure the validity and reliability of information available, and improve their knowledge and awareness of patients’ health behaviors in order to engage this traditionally difficult-to-reach and often high-risk population.

A number of review articles have previously examined the use of social media or social networking sites (SNS) (these terms will be used interchangeably) for health-related research involving the adolescent and young adult population, but each is very focused on defined topic areas, such as specific medical conditions [13], mental health [14], other health-risk behaviors [15,16], and the effectiveness of SNS for health research [17]. To our knowledge, no review articles to date have focused on the use of social media as they relate to adolescent and young adult health care. Therefore, the purpose of this systematic review is to identify research on the use of social media for interacting with adolescents and young adults to achieve positive health outcomes.

## Methods

This study was conducted using the Preferred Reporting Items for Systematic Reviews and Meta Analysis (PRISMA) guidelines [18]. To determine the ways in which social media has been used to interact with adolescents and young adults, we used a systematic approach to retrieve relevant papers from the literature. Articles were selected for this review using the following pre-defined selection criteria (Table 1): (1) involved original research, (2) published in peer-reviewed journals, (3) specified the use of SNS, (4) target population was exclusive to adolescents and/or young adults between the ages of 11-25 years, and (5) written in English. Within MEDLINE / the PubMed electronic database, we performed a search between January 1, 2002 and October 1, 2013. We included keywords (ie, social networking website, Web 2.0, Facebook, Twitter, MySpace), which were selected based on current definitions of social media at the time of this systematic review [15]. We then refined our search using the keyword “health”, as well as keywords synonymous with the adolescent and young adult population (ie, teen, high school student, college student). In addition, we conducted a manual search of articles published within the Journal of Medical Internet Research (JMIR) to retrieve relevant papers. The resulting abstracts were critically reviewed for relevance. We chose to include individuals up to age 25 years in this review for a number of reasons, including the paucity of studies aimed at adolescents under age 18 years and the anticipated similarities in the use of social media, medical implications, and opportunities for intervention.

All full-text articles that met the inclusion criteria were downloaded from PubMed and critically reviewed by two separate researchers (LY, SZ). A checklist for data extraction from the studies was created (Table 2). The purpose of the study, type of social media used, participants and sample size, methodology, and significant findings were summarized. All studies were categorized by methodology (interventional vs observational). Studies were also categorized by the purpose for which social media was employed. These categories were adapted from previously published categorization of uses of social media for health communication [13] and included (1) studies that observed health behaviors by performing content analysis of SNS, assessing SNS use by interview or survey or eliciting reaction to a post on SNS, (2) studies that provided health information, (3) studies that engaged the community, either by facilitating communication with HCPs or creating an online community, and (4) studies that used social media as a means of recruiting participants for clinical research.

Data was extracted independently by the two reviewers and compared. Any discrepancies regarding data categorization were reviewed by a third author (CS) and discussed as a group, after which a consensus was reached and a final database was compiled. As our systematic review focused on the ways in which social media were used, rather than outcomes of its use, further meta-analysis was not performed.

**Table 1:** Inclusion and exclusion criteria for systematic review.

Criteria	
Inclusion criteria	Original research
	Published in peer-reviewed journals
	Involves social media / SNS
	Study population: 11-25 years of age
	Written in English language
Exclusion criteria	Not original research: reviews, editorials, and commentaries
	Methodology or technical papers
	Target population not adolescents or young adults
	Not focused on, or involving, online SNS

**Table 2:** Checklist for data acquisition for papers included in the systematic review.

Data extraction category	Details
Authors	
Title	
Journal	
Year	
Purpose	
Type of social media	MySpace
	Facebook
	YouTube
	Mixed social networking sites
	Other
Target population	
Sample size	
Health issues assessed	Sexual behaviors, sexually transmitted infections
	Alcohol, drugs, or tobacco
	Cyberbullying or sexual predators
	Mental health issues
	Medical diseases
	Other
Outcomes assessed	
Limitations listed in study	
Study results	
Type of study	Observational
	Interventional
Use of social media	Assess or view social media sites
	Assess social media use via survey or interview
	Elicit reaction to postings on social media
	Provide health information or change behaviors via social media sites
	Recruitment through social media
	Improve communication with health care provider via social media
	Create community within social media

## Results

**Figure 1:**
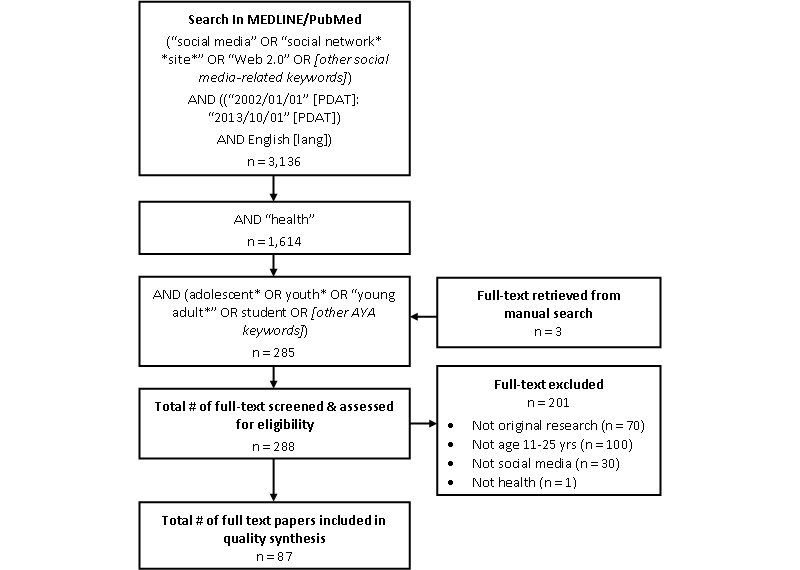
PRISMA flow diagram of selection procedure for systematic review.

We initially identified 3136 studies involving social media; 1614 of these studies were categorized as applying to health medical subject heading (MeSH) terminology and, of these, 288 involved the adolescents and young adult population. Of these studies, 201 were excluded because they were not original research articles, were not specific to adolescents and young adults (ie, included ages outside of those established in our inclusion criteria of 11-25 years old), or did not involve social media (Table 3). The excluded studies are listed in Multimedia Appendix 1. The PRISMA flow diagram is shown in Figure 1.

**Figure 2:**
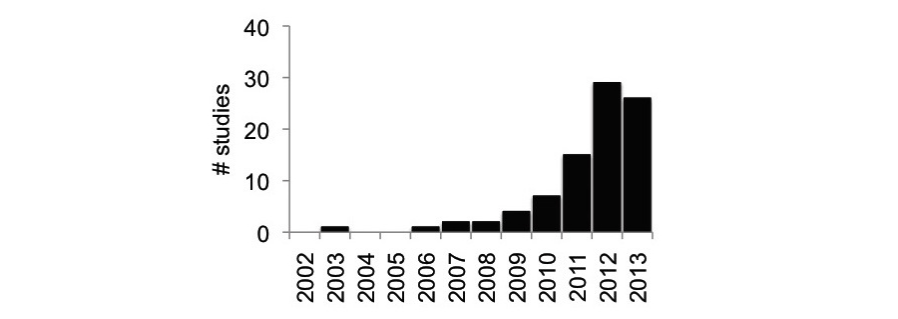
Number of studies meeting criteria for systematic review, by year (studies published through 10/1/2013).

Although our search began around the popularization of Web 2.0 in January 2002, only two studies were published before 2006 [14,17] and the largest number of original research studies were identified as being published in 2012 (n=29) (Figure 2).

Of the 87 studies that were included in our systematic review, 86% (75/87) were observational and 14% (12/87) were interventional. There were four primary ways in which social media were used within these studies: (1) observing adolescent and young adult health behaviors (n=77), (2) providing health information (n=13), (3) engaging the adolescents and young adult community (n=17), and (4) recruiting study participants (n=22). The study of adolescent and young adult health behavior was done in one of three ways: viewing social media sites/content analysis (n=25), assessing social media use by interview or survey (n=46), and eliciting reactions to public posting on social media (n=6). Second, researchers used social media platforms for providing health information (n=13). Third, social media platforms were used to engage the community either by improving communication with HCP (n=7) or leveraging social media to create an online community (n=10). Last, numerous studies used SNS as a means of research recruitment (n=23). Notably, several of the studies incorporated more than one method of using social media (Table 4).

The following types of social media were used: MySpace (n=6), Facebook (n=31), You Tube (n=2), and mixed social media platforms (n=37). A total of 11 studies exclusively involved other types of Web-based platforms, including Bebo, MyLOL.net, patient blogs, email listservs, Web 2.0 portals “Diabit”, “Upopolis”, “NevaEvaLand”, and “Mindcheck.ca” (Table 5).

A range of health care issues are addressed by the studies included in this systematic review. Common themes that arose included high-risk sexual behaviors (n=23), alcohol, tobacco, and other drug use (n=19), cyberbullying or online sexual predators (n=8), and mental health issues (n=18). Several studies focused on specific medical conditions (n=11), such as diabetes, childhood cancers, or other chronic childhood diseases. Other topics (n=12) addressed included organ donation, compulsive Internet use, fitness, anxiety related to dental procedures, feasibility of using social media for research recruitment, social support systems, and general social media use. Several studies addressed overlapping topics. The summary of the findings from these studies is included in Multimedia Appendix 2.

Of the 87 studies, 29 were targeted toward adolescents 11-18 years of age, 53 of the studies included 19-25 year olds, and five of the studies did not define the use of the term adolescent. Of the 12 interventional studies, nine of the studies involved young adults between the ages of 19 and 25 years and only three involved those ≤18 years of age. The content of the studies had notable differences: studies focused on those ≤18 years of age were more likely to focus on cyberbullying/sexual predators and specific medical disease than studies including those of an older age group. Studies involving older adolescents were more apt to focus on sexual behaviors, alcohol, tobacco, and other drug use, and mental health. Studies geared toward younger adolescents were more apt to assess social media use, whereas studies aimed for older adolescents and young adults used social media to provide health information or for research recruitment (Table 6).

Limitations listed within the individual studies were reviewed (several studies reported more than one limitation); 53 of the observational studies reported sampling biases and uncertain generalizability (62%), 34 reported limited number of variables assessed (40%), 30 reported limitations related to self-report (35%), and 20 cited incomplete datasets available due to constraints of SNS (24%). Other limitations included small sample size (n=16, 19%) and inability to assess for causality (n=14, 17%).

**Table 3:** Summary of reasons for exclusion from systematic review (n=288).

Reason for exclusion	n
Not original research: reviews, editorials, and commentaries	59
Methods or technical papers	7
Guidelines	4
Papers not specific to adolescent or young adult ages	100
Papers not clearly involving online social media	30
Papers not relating to human health	1
Total number excluded	201

**Table 4:** Use of social media within research studies.

Social media use		Study
**Observing behaviors (n=77)**		
	Viewing social media sites/content analysis (n=25)	Moreno MA [16,19-25], Egan KG [26], Griffiths R [27], Ridout B [28], Whitehill JM [29], Jenssen BP [30], Pujazon-Zazik MA [31], Marcus MA [32], Lam CG [33], Clerici CA [34], Gao X [35], Egan KG [36], Stokes CE [37], Villiard H [38], Cash SJ [39], Lefkowitz ES [40], Robertson L [41], Brockman LN [42]
	Assessing social media use via interview or survey (n=46)	Ybarra ML [14,43], Pantic I [44], O’Dea B [45,46], Egan KG [26,47], Moreno MA [48,49], Dunlop SM [50], Wang J [51], Lam CG [33], Clerici CA [34], Nordfeldt S [52,53], Divecha Z [54], Bauermeister JA [55], Stoddard SA [56], Lefkowitz ES [40], Yang CC [57], Jelenchick LA [58], van der Velden M [59], Vyas AN [60], Whiteley LB [61], Veinot TC [62], van Rooij AJ [63], Rice E [64,65], Madan G [66], Woolford SJ [67], Juvonen J [68], Perren S [69], Tucker JS [70], Machold C [71], Gowen K [72], Wolniczak I [73], Campisi J [74], Horgan A [75], Dowdell EB [76], Landry M [77], Li TM [2], Struik LL [78], Black SR [79], Selkie EM [80], Veinot TC [81], Pulman A [82]
	Eliciting reaction to public comments (“posts”) on social media (n=6)	Dunlop SM [50], Robertson L [41], Egan K [47], Litt DM [83], Young SD [84], Jones K [85]
Providing health information (n=13)		Lam CG [33], Clerici CA [34], Nordfeldt S [52,53], D’Alessandro AM [86], Moreno MA [24], Hedge KC [87], Rice E [64], Bull SS [88], Jones K [85], Livingston JD [89], Li TM [2], Lu AS [90]
**Engaging a community (n=17)**		
	Improving communication with HCPs (n=7)	Whitehill JM [29], Nordfeldt S [52,53], Hedge KC [87], Jones K [85], Selkie EM [80], Lu AS [90]
	Creating an online community (n=10)	Rice E [64], Nordfeldt S [52,53], Hedge KC [87], Tichon JG [17], Bull SS [88], Livingston JD [89], Jones K [85], van der Velden M [59], Li TM [2]
Research study recruitment (n=23)		Gunasekaran B [91], Jones L [92], Ramo DE [93], Fenner Y [94], Moreno MA [21-24], Whitehill JM [29], Rice E [64], Stoddard SA [56], Bauermeister JA [55], Gamage DG [95], Lord S [96], Ahmed N [97], Chu JL [98], Kraaij V [99], Ezell JM [100], Brockman LN [42], Jones K [85], Struik LL [78], Black SR [79], Veinot TC [81]

**Table 5:** Types of social media used in the studies included in the systematic review.

Social media	Study
MySpace (n=6)	Moreno MA [16,19,20,24,25], Cash SJ [39]
Facebook (n=31)	Egan KG [26,36,47], Ridout B [28], Whitehill JM [29], Ramo DE [93], Moreno MA [21,23] Bauermeister JA [55], Jones L [92], Fenner Y [94], Yang CC [57], Gunasekaran B [91], Madan G [66], Villiard H [38], Lefkowitz ES [40], Brockman LN [42], Wolniczak I [73], Gamage DG [95], Lord S [96], Campisi J [74], Ahmed N [97], Chu JL [98], Black SR [79], Ezell JM [100], Young SD [84], Bull SS [88], Jones K [85], Li TM [2], Litt DM [83], Woolford SJ [67]
YouTube (n=2)	Gao X [35], Clerici CA [34]
Mixed social media (n=37)	Ybarra ML [14,43], Pantic I [44], O’Dea B [45,46], Jenssen BP [30], Moreno MA [22,24,48,49], Dunlop SM [50], Wang J [51], Lam CG [33], D’Alessandro AM [86], Divecha Z [54], Hedge KC [87], Stoddard SA [56], Jelenchick LA [58], Vyas AN [60], Whiteley LB [61], Veinot TC [62], Rice E [64,65], van Rooij AJ [63], Juvonen J [68], Perren S [69], Tucker JS [70], Machold C [71], Nordfeldt S [52], Huang CG [101],Gowen K [72], Horgan A [75], Struik LL [78], Dowdell EB [76], Landry M [77], Selkie EM [80], Pulman A [82], Veinot TC [81]
Other social media (n=11)	Interactive website/portal: myLOL.net: Pujazon-Zazik MA [31], Diabit: Nordfeldt S [53], Upopolis: van der Velden M [59], NevaEvaLand: Stokes C [37], Dutch SNS: Kraaij V [99], mindcheck.ca: Livingston JD [89]
	Blog: Marcus MA [32], Lu AS [90]
	Bebo: Griffiths R [27], Robertson L [41]
	Listserv: sibkids: Tochon JG [17]

**Table 6:** Comparison of studies included in systematic review, by age of target population (n=87).

Study type and content			Exclusively 11-18 years	Including 19-25 years	Unspecified adolescent age
			n (%)		
Observational, n			26	44	5
Interventional, n			3	9	0
**Content of studies**					
	Sex/sexually transmitted infection		5 (16%)	17 (31%)	1 (20%)
	Alcohol, drugs, tobacco		7 (23%)	12 (23%)	0 (0%)
	Cyberbullying/ sexual predators		7 (23%)	1 (2%)	0 (0%)
	Mental health		3 (10%)	15 (29%)	0 (0%)
	Medical disease		6 (19%)	2 (4%)	3 (60%)
	Other		3 (10%)	8 (13%)	1 (20%)
**Use of social media**					
	**Observe behaviors**				
		View social media sites/ content analysis (n=25)	7 (24%)	15 (28%)	3 (60%)
		Assess social media use by interview or survey (n=46)	19 (66%)	26 (49%)	1 (20%)
		Elicit reaction to public posting on social media (n=6)	2 (3%)	4 (14%)	0 (0%)
		Provide health information (n=13)	2 (3%)	9 (17%)	2 (40%)
	**Engage community**				
		Improve communication with HCP (n=7)	2 (7%)	5 (9%)	0 (0%)
		Create online community (n=10)	3 (10%)	6 (11%)	1 (20%)
		Research study recruitment: (n=22)	2 (3%)	21 (40%)	0 (0%)

## Discussion

### Overview

While the use of social media in health care remains in its infancy, a number of themes emerge on how this communication technology is being leveraged to better understand health habits of adolescents and young adults and improve health care delivery to this population. We have found that there are four primary ways in which social media have been used to interact with the adolescent and young adult population.

### Observing Behaviors

Social media can offer powerful insights into the lives of young people. The majority of the studies in this systematic review were observational studies that either analyzed content of social networking profiles of adolescents and young adults, or assessed social media use though questionnaires or interviews of adolescents and young adults regarding their use of social media platforms. Common social behaviors that were observed related to sexual behaviors [26,33-35,44-46,55,57,60,73-75, 77,78,90,93,95-97], alcohol, tobacco, and other drug use [26-30,32,45,47,50,52,57,63,65,68,83,92,94,101], mental health [24,25,31,36,39,48,59,62,64,67,71,85,91,98,99], and online safety [23,37,38,66,81,82,84,89]. By sharing life experiences with the larger public, social media users offer a window into their lives, often revealing the social pressures and expectations they experience. Their postings offer opportunities for HCPs to identify risky health behaviors and health problems that might have been missed during routine health screening, thereby offering opportunities for intervention.

The frequency with which mental health issues are discussed among young people using social media suggest that this offers a potentially fruitful area for the application of social media to help improve the lives of its users. A positive correlation has been found between depressive symptoms and time spent on online social networks [44], suggesting that depression may be common among individuals who spend a significant amount of time on social media. Another study found that over 50% of secondary school students experienced a need for mental health support and 47% believed that SNS could help with these mental problems [45]. Given that adolescents and young adults are already turning to social media for advice and shared experiences, it may behoove HCPs to listen and to seize this opportunity to reach out with accurate information and support.

By enabling individuals to share their thoughts, behaviors, and experiences with a larger audience, social media can also contribute to the establishment of social norms leading to the creation of pressures to “fit in” among adolescents and young adults. A well-known problem, confirmed by analysis of social media content, is the high prevalence of alcohol use among adolescents and young adults: 25% of teenagers ages 16-17 years old [16], 56% of 17-20 year olds [19], and 85% of college students [26] display references to alcohol on social media. The high prevalence of alcohol references on these “intoxigenic” digital spaces [27], a term used by one author to describe pro-alcohol sentiments on social media, leads to the normalization of alcohol use. Given that 60% of college students report potentially problematic alcohol use [28], such normalization of high-risk behavior online adds to the already daunting challenge that HCPs face in counseling against alcohol abuse. However, the use of social media by providers for this purpose may be limited by its perceived acceptability by adolescent and young adult users. One study investigating preferred means of communication regarding mental health issues found that adolescents did not feel comfortable having an unknown HCP screen their social media sites, and described having a stranger screening their Facebook pages for signs of depression as “creepy” [29].

The normalization of high-risk behaviors is not limited to alcohol alone. The use of tobacco and other drugs, violence, sexual behavior, and even suicidality are also commonly displayed on social media platforms. Tobacco imagery is frequently shared on social media [30] and 9% of teenagers aged 16-17 years describe or display cigarette use on their SNS profiles [16]; 15-24% of adolescents also displayed sexual references [20,31]. Such postings have particularly concerning implications, as they may increase sexual expectations when adolescents are using social media to evaluate potential partners [49]. Even suicidality has been found to be impacted by social media through the availability of suicide stories [50].

Online safety is a major concern among studies involving social media: 9% of children ages 10-17 years report having been harassed online over the course of a year [14] and 4% received unwanted sexual solicitation [43]. This has important implications, not just for safety, but also for mental health as cyber-bully victims have higher depression rates when compared to traditional “offline” bully-victims [51]. Adding to the potential risk is the fact that even potentially sensitive behaviors are typically not posted anonymously: one study found that 97% of SNS contained personal identifiers, such as including a profile photo, full name, and hometown [32]. However, privacy settings within different SNS varies and information that may be viewed publically may change over time.

### Providing Health Information

A number of studies have used social media platforms as channels to provide health information to educate and invoke behavior change among young people. These platforms have tended to be disease-specific, providing information on childhood cancers [33,34], diabetes [53], and organ-donation [86]. Although it has been suggested that social media may not be a preferred method of contact regarding health information [54], most such studies nonetheless demonstrated a positive impact on their target audiences. For example, an intensive organ donation program utilizing multiple social media platforms to provide organ donation statistics and information was able to increase organ donor registration by 28% [86]. Another study, targeting adolescents at high risk for sexually transmitted diseases, found that brief preventive counseling in the form of a message sent by social media reduced the display of “risky” behaviors online [24].

### Engaging Adolescents and Young Adults

Social media also provides an opportunity for the health care community to become involved in discussions with adolescents and young adults, thereby engaging them in ways not possible within the traditional office setting. This may indeed be the true “gold mine” of incorporating social media into health care for this population. As supported by our review of the literature, researchers have only recently begun exploring ways to reach out to adolescents and young adults through social media in the hopes of creating online communities to improve patient-provider communication. Studies performed to date have primarily assessed the acceptability of using social media through observational studies to interact with young people and have achieved mixed results, likely reflecting our collective lack of experience with the use of social media for this purpose. For example, a study that invited members to join an interactive Web 2.0 portal consisting of an extensive library of health education information, in the form of text, videos, and simulation software, social networking capability through message boards and blogs, services for renewing medications and scheduling appointments, and sending questions to the medical team, found that participants welcomed this type of health-related community as a source of information and support [53]. Nevertheless, engagement with the Web 2.0 portal was hindered by the lack of frequently updated information and complicated log-in procedures [53]. Clearly, a social media platform that fails to sustain user engagement is unlikely to be an effective means of improving health care outcomes in the long term [87].

### Recruiting Adolescents and Young Adults for Research

Beyond engaging adolescents and young adults in discussions about health, social media can also be used in research recruitment efforts. A number of studies identified participants from their postings on social media and subsequently approached them about participating [21-23,29,55,56,93]. Another study was able to leverage social media to locate study participants who would have otherwise been lost to follow-up [92]. In addition to being a cost-effective and efficient means of research recruitment, social media enables researchers to reach a demographically representative sample of adolescents and young adults, including those traditionally underrepresented in research (eg, high-risk individuals and those living in rural communities), and also by providing real-time monitoring of recruitment efforts [94]. Such findings suggest that recruitment of adolescents and young adults for research studies may be achieved more effectively and efficiently through social media channels.

### Barriers to Using Social Media

Despite the wealth of opportunities, there remains concern and potential barriers to using social media for health care applications in the adolescent and young adult population. Privacy and confidentiality issues are a concern, particularly when discussing sensitive or stigmatized health topics online through non-secured formats, and young people have expressed a preference for accessing credible health-related information anonymously [80]. Because adolescents generally prefer to seek help from their peers and people that they know rather than from HCPs and strangers, developing an acceptable way to leverage social media for health care purposes may be difficult [23,29]. In their efforts to interact with young people over social media in ways that will engage them, HCPs must be mindful of maintaining professional boundaries and patient privacy. Another concern is that HCPs would be expected to keep up with this ever-changing, fast-paced dialogue on social media that can evolve and spread quickly, with potentially dire consequences. For example, in the case of suicide contagion, it may not be possible for providers to recognize the problem and intervene quickly enough to prevent adolescents and young adults from harm. Furthermore, statements made on social media may not reflect the writer’s actual state of health or behaviors, particularly if adolescents and young adults exaggerate or falsify information that they share over social media as a result of social desirability bias. It is important to also note that the majority of studies that we reviewed focused on publicly available content on social media, and what teens share openly may not fully reflect the true extent of their behaviors in their day-to-day lives. Last, because of the open nature of social media and the potential for posting of exaggerated, falsified, or untrue statements on these platforms, the credibility and trustworthiness of the posted content will remain an issue, unless there are systems in place to monitor the quality and content of information on social media platforms.

### Limitations

The strengths of our paper lie in the comprehensive and systematic approach we took to review the literature, along with our deliberate and detailed approach to reviewing each full-text article. Nevertheless, there are limitations to this systematic review that warrant considering. First, it is possible that, despite our attempts to capture all pertinent articles through the use of numerous carefully selected search terms, some relevant studies may have unintentionally been excluded. Furthermore, because many social media platforms (including Twitter, Pinterest, and Flickr) do not include documentation of age, many of the observational studies on these platforms were excluded as the age of the study population could not be confirmed. Another potential limitation of the study is in our inclusion criteria for age. We chose to include research involving adolescents as young as 11 years old up to young, college-aged adults of ages up to 25 years due to similarities in the health risks and concerns, reported behaviors, and patterns of social media use. This broader age range provided a greater volume of studies, which we believe offered more comprehensive insight into the potential uses of social media to impact the health of young people. Furthermore, it is possible that studies pertinent to this review may have been missed as a result of keywords used in the article selection process. However, trends for use of SNS within adolescents and young adults were observed, as described above.

### Future Directions

Social media and social networking platforms are relatively novel ways of communication, driven primarily by young people, which have been growing and changing ever since their emergence in the early 21^st^ century. Currently, over 70% of adults obtain health information predominantly online [102], but given the growing popularity of social media, it has the potential of becoming a more significant source of online health information in the coming years. Users of social media and those in other fields, such as the advertising industry, understand how the medium can be leveraged for the sharing of information, but the medical and scientific community has been slow to embrace these technologies. Ironically, many health care providers overlook the fact that when they themselves search for health information online on search engines, they often find themselves on Wikipedia, which itself is a social media platform.

Understanding how individuals engage on SNS and consume information will enable HCPs and health care provider organizations to make their content and patient engagement strategies more online-friendly. The number of people using social networking platforms daily continues to grow steadily and this ubiquity can help HCPs engage with patients on a platform they may already be using. For example, communicating with patients about office appointments, lab tests, or even allowing them to ask questions and share content directly through SNS could greatly facilitate patient-provider communication and increase patients’ participation in their own care. Additionally, with the emergence of mobile technology, SNS are becoming increasingly “real time”, immediate, and local. The combination of SNS with mobile technology makes it possible to learn about patient behaviors and daily habits, and to intervene with relevant and timely messaging, coaching, and interventions. Furthermore, computer-generated predictive analytics could be established to screen SNS for keywords or images associated with health care-related issues, automating the process of SNS surveillance in a more patient-accepted format, which may feel less “creepy” to adolescents and young adults while still capturing important opportunities for positive intervention. In the future, the rich data available through SNS and mobile technology may enable HCPs to become more proactive about health care delivery.

### Conclusions

Although many HCPs remain timid about the use of social media in the care of patients, this review demonstrates that social media is already being used for a variety of purposes and in a number of different ways to engage, educate, and improve the health of its users. Most studies done to date have been observational in nature, examining how adolescents and young adults communicate on social media and the resulting implications on their health. Although these explorations are essential, further exploration and development of these strategies into building effective interventions that can positively impact the health of young people is warranted. One of the greatest challenges in harnessing social media is the constant and rapid pace of evolution, including the continual development of new technologies and the ever-changing popularity and adoption of specific platforms among different user demographics. In order to stay on top of this rapidly evolving field, ongoing study of the use of SNS by adolescents and young adults will be critical. Further research is necessary to establish whether social media can be an effective tool to help achieve positive health outcomes in the adolescent and young adult population.

## Multimedia Appendix 1

List of excluded studies (n=201).

## Multimedia Appendix 2

Summary table of studies included in systematic review.

## Abbreviations

HCP: health care providers
PRISMA: Preferred Reporting Items for Systematic Reviews and Meta Analysis
SNS: social networking sites
