# Supplementary material for: “Friending” Teens: Systematic Review of Social Media in Adolescent and Young Adult Health Care
Source: J Med Internet Res. 2015 Jan 5;17(1):e4. doi: 10.2196/jmir.3692 (PMC4376201; doi:10.2196/jmir.3692)
Supplement: Supplementary file 1 [file medinform_v17i1e297_app1.pdf]

Multimedia Appendix 1. List of excluded studies (n=201).

Reviews, Editorials & Commentaries (n=59)

1. Nickels A, Dimov V. Innovations in technology: social media and mobile technology in the care of adolescents with asthma. Curr Allergy Asthma Rep. 2012 Dec;12(6):607-12. doi: 10.1007/s11882-012-0299-7. Review.
2. Guse K, Levine D, Martins S, Lira A, Gaarde J, Westmorland W, Gilliam M. Interventions using new digital media to improve adolescent sexual health: a systematic review. J Adolesc Health. 2012 Dec;51(6):535-43. doi: 10.1016/j.jadohealth.2012.03.014. Epub 2012 May 5. Review.
3. Berry DM, Bass CP. Successfully recruiting, surveying, and retaining college students: a description of methods for the Risk, Religiosity, and Emerging Adulthood Study. Res Nurs Health. 2012 Dec;35(6):659-70. doi: 10.1002/nur.21498. Epub 2012 Jul 2.
4. von Muhlen M, Ohno-Machado L. Reviewing social media use by clinicians. J Am Med Inform Assoc. 2012 Sep-Oct;19(5):777-81. doi: 10.1136/amiajnl-2012-000990. Epub 2012 Jul 3. Review.
5. Sawyer SM, Afifi RA, Bearinger LH, Blakemore SJ, Dick B, Ezeh AC, Patton GC. Adolescence: a foundation for future health. Lancet. 2012 Apr 28;379(9826):1630-40. doi: 10.1016/S0140-6736(12)60072-5. Epub 2012 Apr 25.
6. Hyden C, Cohall A. Innovative approaches to using new media and technology in health promotion for adolescents and young adults. Adolesc Med State Art Rev. 2011 Dec;22(3):498-520, xi-xii.
7. Meloy JR, O'Toole ME. The concept of leakage in threat assessment. Behav Sci Law. 2011 Jul-Aug;29(4):513-27. doi: 10.1002/bsl.986. Epub 2011 Jun 28.
8. Goodman J, Wennerstrom A, Springgate BF. Participatory and social media to engage youth: from the Obama campaign to public health practice. Ethn Dis. 2011 Summer;21(3 Suppl 1):S1-94-9.
9. O'Keeffe GS, Clarke-Pearson K; Council on Communications and Media. The impact of social media on children, adolescents, and families. Pediatrics. 2011 Apr;127(4):800-4. doi: 10.1542/peds.2011-0054. Epub 2011 Mar 28.
10. Messina ES, Iwasaki Y. Internet use and self-injurious behaviors among adolescents and young adults: an interdisciplinary literature review and implications for health professionals. Cyberpsychol Behav Soc Netw. 2011 Mar;14(3):161-8. doi: 10.1089/cyber.2010.0025. Epub 2010 Aug 2. Review.
11. Treadgold CL, Kuperberg A. Been there, done that, wrote the blog: the choices and challenges of supporting adolescents and young adults with cancer. J Clin Oncol. 2010 Nov 10;28(32):4842-9. doi: 10.1200/JCO.2009.23.0516. Epub 2010 Mar 29. Review.
12. van Manen M. The pedagogy of Momus technologies: Facebook, privacy, and online intimacy. Qual Health Res. 2010 Aug;20(8):1023-32. doi: 10.1177/1049732310364990. Epub 2010 Mar 24.
13. Pujazon-Zazik M, Park MJ. To tweet, or not to tweet: gender differences and potential positive and negative health outcomes of adolescents' social internet use. Am J Mens Health. 2010 Mar;4(1):77-85. doi: 10.1177/1557988309360819. Review.
14. Griffiths MD, Parke J. Adolescent gambling on the internet: a review. Int J Adolesc Med Health. 2010 Jan-Mar;22(1):59-75. Review.
15. Kind T. The Internet as an adjunct for pediatric primary care. Curr Opin Pediatr. 2009 Dec;21(6):805-10. doi: 10.1097/MOP.0b013e328331e7b4. Review.
16. Williams AL, Merten MJ. A review of online social networking profiles by adolescents: implications for future research and intervention. Adolescence. 2008 Summer;43(170):253-74.
17. Moreno MA, Fost NC, Christakis DA. Research ethics in the MySpace era. Pediatrics. 2008 Jan;121(1):157-61. doi: 10.1542/peds.2007-3015. Review.
18. Kirschner KL, Brashler R, Savage TA. Ashley X. Am J Phys Med Rehabil. 2007 Dec;86(12):1023-9. Review.
19. Norman CD, Skinner HA. Engaging youth in e-health promotion: lessons learned from a decade of TeenNet research. Adolesc Med State Art Rev. 2007 Aug;18(2):357-69, xii. Review.

20. Jones E, Sinclair JM, Holt RI, Barnard KD. Social networking and understanding alcohol-associated risk for people with type 1 diabetes: friend or foe? Diabetes Technol Ther. 2013 Apr;15(4):308-14. doi: 10.1089/dia.2012.0327. Epub 2013 Feb 19. Review.
21. Chisholm JF. Cyberspace violence against girls and adolescent females. Ann N Y Acad Sci. 2006 Nov;1087:74-89. Review.
22. Genuis SJ, Genuis SK. Internet interactions: adolescent health and cyberspace. Can Fam Physician. 2005 Mar;51:329-31, 334-6. English, French. No abstract available.
23. Amicizia D, Domnich A, Gasparini R, Bragazzi NL, Lai PL, Panatto D. An overview of current and potential use of information and communication technologies for immunization promotion among adolescents. Hum Vaccin Immunother. 2013 Aug 17;9(12). [Epub ahead of print]
24. Stockwell MS, Fiks AG. Utilizing health information technology to improve vaccine communication and coverage. Hum Vaccin Immunother. 2013 Aug;9(8):1802-11. doi: 10.4161/hv.25031. Epub 2013 Jun 4. Review.
25. Park BK, Calamaro C. A systematic review of social networking sites: innovative platforms for health research targeting adolescents and young adults. J Nurs Scholarsh. 2013 Sep;45(3):256-64. doi: 10.1111/jnu.12032. Epub 2013 May 15.
26. Kelly L. I know it shouldn't but it still hurts" bullying and adults: implications and interventions for practice." Nurs Clin North Am. 2011 Dec;46(4):423-9, v-vi. doi: 10.1016/j.cnur.2011.08.003. Epub 2011 Oct 17.
27. Weaver B, Lindsay B, Gitelman B. Communication technology and social media: opportunities and implications for healthcare systems. Online J Issues Nurs. 2012 Sep 30;17(3):3.
28. Genuis SJ, Genuis SK. Implications of cyberspace communication: a role for physicians. South Med J. 2005 Apr;98(4):451-5; quiz 456-7, 477.
29. Lemma A. An order of pure decision: growing up in a virtual world and the adolescent's experience of being-in-a-body. J Am Psychoanal Assoc. 2010 Aug;58(4):691-714. doi: 10.1177/0003065110385576.
30. Andrew S, Cleary M, Jackson D. Facing a new frontier: safety in cyberspace and challenges for nursing. J Psychosoc Nurs Ment Health Serv. 2012 Aug;50(8):4-5. doi: 10.3928/02793695-20120703-05. No abstract available.
31. Smith JC, Mailman T, MacDonald NE. How to get and get rid of gonorrhea. Adv Exp Med Biol. 2013;764:219-39. Review.
32. Vance K, Howe W, Dellavalle RP. Social internet sites as a source of public health information. Dermatol Clin. 2009 Apr;27(2):133-6, vi. doi: 10.1016/j.det.2008.11.010. Review.
33. Annang L, Muilenburg JL, Strasser SM. Virtual worlds: taking health promotion to new levels. Am J Health Promot. 2010 May-Jun;24(5):344-6, iii. doi: 10.4278/ajhp.08042236.
34. Birbal R, Maharajh HD, Birbal R, Clapperton M, Jarvis J, Ragoonath A, Uppalapati K. Cybersuicide and the adolescent population: challenges of the future? Int J Adolesc Med Health. 2009 Apr-Jun;21(2):151-9. Review.
35. Preliminary experience with social media for community consultation and public disclosure in exception from informed consent trials. Circulation. 2013 Jul 16;128(3):267-70. doi: 10.1161/CIRCULATIONAHA.113.002390.
36. Williams S. Action needed to combat food and drink companies' social media marketing to adolescents. Perspect Public Health. 2013 May;133(3):146-7. doi: 10.1177/1757913913484871.
37. Prasad B. Social media, health care, and social networking. Gastrointest Endosc. 2013 Mar;77(3):492-5. doi: 10.1016/j.gie.2012.10.026.
38. Stanwell-Smith R. Editorial: getting into blogs and other teen concerns. Perspect Public Health. 2013 Mar;133(2):74. doi: 10.1177/1757913913476618.
39. Bradshaw D, Hughes A, Day S. A novel service promotion campaign using the social media site Facebook. Sex Transm Infect. 2013 Mar;89(2):104. doi: 10.1136/sextrans-2012-050877. Epub 2013 Jan 18.
40. Collier B, Blackstone SW, Taylor A. Communication access to businesses and organizations for people with complex communication needs. Augment Altern Commun. 2012 Dec;28(4):205-18. doi: 10.3109/07434618.2012.732611. Epub 2012 Nov 13.

41. Moreno MA, Whitehill JM. New media, old risks: toward an understanding of the relationships between online and offline health behavior. Arch Pediatr Adolesc Med. 2012 Sep;166(9):868-9. doi: 10.1001/archpediatrics.2012.1320.
42. Grant MJ. Health, sport and nutritional information: tailoring your approach. Health Info Libr J. 2012 Jun;29(2):87-9. doi: 10.1111/j.1471-1842.2012.00991.
43. Rice E, Karnik NS. Network science and social media. J Am Acad Child Adolesc Psychiatry. 2012 Jun;51(6):563-5. doi: 10.1016/j.jaac.2012.02.008.
44. Moreno MA, Kolb J. Social networking sites and adolescent health. Pediatr Clin North Am. 2012 Jun;59(3):601-12, vii. doi: 10.1016/j.pcl.2012.03.023. Epub 2012 Apr 18.
45. O'Keeffe GS. Overview: new media. Pediatr Clin North Am. 2012 Jun;59(3):589-600, vii. doi: 10.1016/j.pcl.2012.03.024.
46. O'Sullivan LF. Open to the public: how adolescents blur the boundaries online between the private and public spheres of their lives. J Adolesc Health. 2012 May;50(5):429-30. doi: 10.1016/j.jadohealth.2012.03.001.
47. Casacchia M, Pollice R, Roncone R. The narrative epidemiology of L'Aquila 2009 earthquake. Epidemiol Psychiatr Sci. 2012 Mar;21(1):13-21.
48. Kerson TS. Epilepsy postings on YouTube: exercising individuals' and organizations' right to appear. Soc Work Health Care. 2012;51(10):927-43. doi: 10.1080/00981389.2012.712634.
49. Bull SS, Breslin LT, Wright EE, Black SR, Levine D, Santelli JS. Case study: An ethics case study of HIV prevention research on Facebook: the Just/Us study. J Pediatr Psychol. 2011 Nov-Dec;36(10):1082-92. doi: 10.1093/jpepsy/jsq126. Epub 2011 Feb 3.
50. Crutzen R, De Nooijer J. Intervening via chat: an opportunity for adolescents' mental health promotion? Health Promot Int. 2011 Jun;26(2):238-43. doi: 10.1093/heapro/daq062. Epub 2010 Oct 21.
51. Solecki S, Goldschmidt K. Adolescents texting and twittering: the flash mob phenomena. J Pediatr Nurs. 2011 Apr;26(2):167-9. doi: 10.1016/j.pedn.2010.12.013.
52. Spriggs M. Consent in cyberspace: Internet-based research involving young people. Monash Bioeth Rev. 2009 Dec;28(4):32.1-15.
53. Mgweba L, Dlamini S, Kassim J, Planting T, Smith D. Facebook is smoking. S Afr Med J. 2009 Nov;99(11):768, 770.
54. Mitchell KJ, Ybarra M. Social networking sites: finding a balance between their risks and benefits. Arch Pediatr Adolesc Med. 2009 Jan;163(1):87-9. doi: 10.1001/archpediatrics.2008.534.
55. Klein DA, Myhre KK, Ahrendt DM. Bullying among adolescents: a challenge in primary care. Am Fam Physician. 2013 Jul 15;88(2):87-92. No abstract available.
56. Varley CK. Perspectives of adolescents with attention-deficit hyperactivity disorder do matter. J Adolesc Health. 2011 Jul;49(1):1-2. doi: 10.1016/j.jadohealth.2011.05.003. No abstract available.
57. Mackey TK, Liang BA, Strathdee SA. Digital social media, youth, and nonmedical use of prescription drugs: the need for reform. J Med Internet Res. 2013 Jul 26;15(7):e143. doi: 10.2196/jmir.2464.
58. Danovitch I, Gorelick DA. State of the art treatments for cannabis dependence. Psychiatr Clin North Am. 2012 Jun;35(2):309-26. doi: 10.1016/j.psc.2012.03.003. Epub 2012 Apr 10. Review.
59. Ogburn KM, Messias E, Buckley PF. New-age patient communications through social networks. Gen Hosp Psychiatry. 2011 Mar-Apr;33(2):200.e1-3. doi: 10.1016/j.genhosppsych.2010.08.006. Epub 2011 Jan 13.

Methods or Technical Papers (n=7)

1. Gabarron E, Serrano JA, Wynn R, Armayones M. Avatars using computer/smartphone mediated communication and social networking in prevention of sexually transmitted diseases among North-Norwegian youngsters. BMC Med Inform Decis Mak. 2012 Oct 30;12:120. doi: 10.1186/1472-6947-12-120.
2. Norman CD, Yip AL. eHealth promotion and social innovation with youth: using social and visual media to engage diverse communities. Stud Health Technol Inform. 2012;172:54-70.

3. Levine D, Madsen A, Wright E, Barar RE, Santelli J, Bull S. Formative research on MySpace: online methods to engage hard-to-reach populations. J Health Commun. 2011 Apr;16(4):448-54. doi: 10.1080/10810730.2010.546486
4. Timpka T, Eriksson H, Ludvigsson J, Ekberg J, Nordfeldt S, Hanberger L. Web 2.0 systems supporting childhood chronic disease management: a pattern language representation of a general architecture. BMC Med Inform Decis Mak. 2008 Nov 28;8:54. doi: 10.1186/1472-6947-8-54.
5. Long MD, Kappelman MD, Martin CF, Lewis JD, Mayer L, Kinneer PM, Sandler RS. Development of an internet-based cohort of patients with inflammatory bowel diseases (CCFA Partners): methodology and initial results. Inflamm Bowel Dis. 2012 Nov;18(11):2099-106. doi: 10.1002/ibd.22895. Epub 2012 Jan 27.
6. Killackey E, Anda AL, Gibbs M, Alvarez-Jimenez M, Thompson A, Sun P, Baksheev GN. Using internet enabled mobile devices and social networking technologies to promote exercise as an intervention for young first episode psychosis patients. BMC Psychiatry. 2011 May 12;11:80. doi: 10.1186/1471-244X-11-80.
7. Norman CD, Charnaw-Burger J, Yip AL, Saad S, Lombardo C. Designing health innovation networks using complexity science and systems thinking: the CoNEKTR model. J Eval Clin Pract. 2010 Oct;16(5):1016-23. doi: 10.1111/j.1365-2753.2010.01534.x.

Guidelines (n=4)

1. Rowel R, Sheikhattari P, Barber TM, Evans-Holland M. Introduction of a guide to enhance risk communication among low-income and minority populations: a grassroots community engagement approach. Health Promot Pract. 2012 Jan;13(1):124-32. doi: 10.1177/1524839910390312. Epub 2011 Jul 7
2. Gualtieri L. The potential for social media to educate farm families about health and safety for children. J Agromedicine. 2012;17(2):232-9. doi: 10.1080/1059924X.2012.658268.
3. Ekberg J, Ericson L, Timpka T, Eriksson H, Nordfeldt S, Hanberger L, Ludvigsson J. Web 2.0 systems supporting childhood chronic disease management: design guidelines based on information behaviour and social learning theories. J Med Syst. 2010 Apr;34(2):107-17.
4. Impact of media on children. Aust Nurs J. 2012 May;19(10):42-3. No abstract available.

Papers not specific to Adolescents or Young Adult age group (n=100)

1. Thornton LK, Baker AL, Johnson MP, Lewin T. Perceived risk associated with tobacco, alcohol and cannabis use among people with and without psychotic disorders. Addict Behav. 2013 Jun;38(6):2246-51. doi: 10.1016/j.addbeh.2013.02.003. Epub 2013 Feb 18.
2. Hebden L, Balestracci K, McGeechan K, Denney-Wilson E, Harris M, Bauman A, Allman-Farinelli M. 'TXT2BFiT' a mobile phone-based healthy lifestyle program for preventing unhealthy weight gain in young adults: study protocol for a randomized controlled trial. Trials. 2013 Mar 18;14:75. doi: 10.1186/1745-6215-14-75.
3. Syed-Abdul S, Fernandez-Luque L, Jian WS, Li YC, Crain S, Hsu MH, Wang YC, Khandregzen D, Chuluunbaatar E, Nguyen PA, Liou DM. Misleading health-related information promoted through video-based social media: anorexia on YouTube. J Med Internet Res. 2013 Feb 13;15(2):e30. doi: 10.2196/jmir.2237.
4. Pedrana A, Hellard M, Gold J, Ata N, Chang S, Howard S, Asselin J, Ilic O, Batrouney C, Stoove M. Queer as F**k: reaching and engaging gay men in sexual health promotion through social networking sites. J Med Internet Res. 2013 Feb 7;15(2):e25. doi: 10.2196/jmir.2334.
5. Apatu EJ, Alperin M, Miner KR, Wiljer D. A drive through Web 2.0: an exploration of driving safety promotion on Facebook™. Health Promot Pract. 2013 Jan;14(1):88-95. doi: 10.1177/1524839911405845. Epub 2011 May 2.
6. Wright KB, Rosenberg J, Egbert N, Ploeger NA, Bernard DR, King S. Communication competence, social support, and depression among college students: a model of facebook and face-to-face support network influence. J Health Commun. 2013;18(1):41-57. doi: 10.1080/10810730.2012.688250. Epub 2012 Oct 3.

7. Lohse B. Facebook is an effective strategy to recruit low-income women to online nutrition education. J Nutr Educ Behav. 2013 Jan-Feb;45(1):69-76. doi: 10.1016/j.jneb.2012.06.006
8. Shaeer O, Shaeer K. The Global Online Sexuality Survey (GOSS): the United States of America in 2011. Chapter I: erectile dysfunction among English-speakers. J Sex Med. 2012 Dec;9(12):3018-27. doi: 10.1111/j.1743-6109.2012.02976.x. Epub 2012 Oct 22.
9. D'Alessandro AM, Peltier JW, Dahl AJ. Use of social media and college student organizations to increase support for organ donation and advocacy: a case report. Prog Transplant. 2012 Dec;22(4):436-41.
10. Nordfeldt S, Ängarne-Lindberg T, Berterö C. To use or not to use--practitioners' perceptions of an open web portal for young patients with diabetes. J Med Internet Res. 2012 Nov 9;14(6):e154. doi: 10.2196/jmir.1987.
11. Liang BA, Mackey TK, Lovett KM. Suspect online sellers and contraceptive access. Contraception. 2012 Nov;86(5):551-6. doi: 10.1016/j.contraception.2012.04.011. Epub 2012 May 26.
12. Ressler PK, Bradshaw YS, Gualtieri L, Chui KK. Communicating the experience of chronic pain and illness through blogging. J Med Internet Res. 2012 Oct 23;14(5):e143. doi: 10.2196/jmir.2002.
13. Callander D, Holt M, Newman CE. Just a preference: racialised language in the sex-seeking profiles of gay and bisexual men. Cult Health Sex. 2012 Oct;14(9):1049-63. doi: 10.1080/13691058.2012.714799. Epub 2012 Sep 4.
14. Cucchetti A, Zanello M, Bigonzi E, Pellegrini S, Cescon M, Ercolani G, Mazzotti F, Pinna AD. The use of social networking to explore knowledge and attitudes toward organ donation in Italy. Minerva Anestesiol. 2012 Oct;78(10):1109-16.
15. Graham AL, Fang Y, Moreno JL, Streiff SL, Villegas J, Muñoz RF, Tercyak KP, Mandelblatt JS, Vallone DM. Online advertising to reach and recruit Latino smokers to an internet cessation program: impact and costs. J Med Internet Res. 2012 Aug 27;14(4):e116. doi: 10.2196/jmir.2162.
16. Kelly J, Fealy GM, Watson R. The image of you: constructing nursing identities in YouTube. J Adv Nurs. 2012 Aug;68(8):1804-13. doi: 10.1111/j.1365-2648.2011.05872.x. Epub 2011 Nov 9.
17. Thomas M, Mackay S, Salsbury D. Exposure to fire setting behavior on YouTube. J Adolesc Health. 2012 Jul;51(1):99-100. doi: 10.1016/j.jadohealth.2011.11.018. Epub 2012 Feb 22.
18. Carter B, Coad J, Bray L, Goodenough T, Moore A, Anderson C, Clinchant A, Widdas D. Home-based care for special healthcare needs: community children's nursing services. Nurs Res. 2012 Jul-Aug;61(4):260-8. doi: 10.1097/NNR.0b013e31825b6848.
19. Frew PM, Painter JE, Hixson B, Kulb C, Moore K, del Rio C, Esteves-Jaramillo A, Omer SB. Factors mediating seasonal and influenza A (H1N1) vaccine acceptance among ethnically diverse populations in the urban south. Vaccine. 2012 Jun 13;30(28):4200-8. doi: 10.1016/j.vaccine.2012.04.053. Epub 2012 Apr 23.
20. Shindel AW, Rowen TS, Lin TC, Li CS, Robertson PA, Breyer BN. An Internet survey of demographic and health factors associated with risk of sexual dysfunction in women who have sex with women. J Sex Med. 2012 May;9(5):1261-71. doi: 10.1111/j.1743-6109.2012.02659.x. Epub 2012 Feb 29.
21. Seidenberg AB, Rodgers EJ, Rees VW, Connolly GN. Youth access, creation, and content of smokeless tobacco ("dip") videos in social media. J Adolesc Health. 2012 Apr;50(4):334-8. doi: 10.1016/j.jadohealth.2011.09.003. Epub 2011 Nov 4.
22. Henderson EM, Rosser BA, Keogh E, Eccleston C. Internet sites offering adolescents help with headache, abdominal pain, and dysmenorrhoea: a description of content, quality, and peer interactions. J Pediatr Psychol. 2012 Apr;37(3):262-71. doi: 10.1093/jpepsy/jsr100. Epub 2011 Dec 12.
23. Fisher J, Clayton M. Who gives a tweet: assessing patients' interest in the use of social media for health care. Worldviews Evid Based Nurs. 2012 Apr;9(2):100-8. doi: 10.1111/j.1741-6787.2012.00243.x. Epub 2012 Mar 20.
24. Henry RK, Molnar A, Henry JC. A survey of US dental practices' use of social media. J Contemp Dent Pract. 2012 Mar 1;13(2):137-41.
25. Cancer survivor identity shared in a social media intervention. J Pediatr Oncol Nurs. 2012 Mar-Apr;29(2):80-91. doi: 10.1177/1043454212438964.

26. Gass K, Hoff CC, Stephenson R, Sullivan PS. Sexual agreements in the partnerships of internet-using men who have sex with men. AIDS Care. 2012;24(10):1255-63. doi: 10.1080/09540121.2012.656571. Epub 2012 Mar 1.
27. Omurtag K, Jimenez PT, Ratts V, Odem R, Cooper AR. The ART of social networking: how SART member clinics are connecting with patients online. Fertil Steril. 2012 Jan;97(1):88-94. doi: 10.1016/j.fertnstert.2011.10.001. Epub 2011 Nov 14.
28. Wagenaar BH, Christiansen-Lindquist L, Khosropour C, Salazar LF, Benbow N, Prachand N, Sineath RC, Stephenson R, Sullivan PS. Willingness of US men who have sex with men (MSM) to participate in Couples HIV Voluntary Counseling and Testing (CVCT). PLoS One. 2012;7(8):e42953. doi: 10.1371/journal.pone.0042953. Epub 2012 Aug 14.
29. Ruthig JC, Holfeld B, Hanson BL. The role of positive thinking in social perceptions of cancer outcomes.
30. Leighton JW, Valverde K, Bernhardt BA. The general public's understanding and perception of direct-to-consumer genetic test results. Public Health Genomics. 2012;15(1):11-21. doi: 10.1159/000327159. Epub 2011 Jun 30.
31. Stroever SJ, Mackert MS, McAlister AL, Hoelscher DM. Using social media to communicate child health information to low-income parents. Prev Chronic Dis. 2011 Nov;8(6):A148. Epub 2011 Oct 17.
32. Simunaniemi AM, Sandberg H, Andersson A, Nydahl M. Laypeople blog about fruit and vegetables for self-expression and dietary influence. Health Commun. 2011 Oct;26(7):621-30. doi: 10.1080/10410236.2011.561520. Epub 2011 Jul 17.
33. Jent JF, Eaton CK, Merrick MT, Englebert NE, Dandes SK, Chapman AV, Hershorin ER. The decision to access patient information from a social media site: what would you do? J Adolesc Health. 2011 Oct;49(4):414-20. doi: 10.1016/j.jadohealth.2011.02.004. Epub 2011 May 24.
34. Baptist AP, Thompson M, Grossman KS, Mohammed L, Sy A, Sanders GM. Social media, text messaging, and email-preferences of asthma patients between 12 and 40 years old. J Asthma. 2011 Oct;48(8):824-30. doi: 10.3109/02770903.2011.608460. Epub 2011 Aug 24.
35. Garcia-Romero MT, Prado F, Dominguez-Cherit J, Hojyo-Tomomka MT, Arenas R. Teledermatology via a social networking web site: a pilot study between a general hospital and a rural clinic. Telemed J E Health. 2011 Oct;17(8):652-5. doi: 10.1089/tmj.2011.0038. Epub 2011 Jul 26.
36. Casale S, Fioravanti G. Psychosocial correlates of internet use among Italian students. Int J Psychol. 2011 Aug;46(4):288-98. doi: 10.1080/00207594.2010.541256.
37. Giordano C, Giordano C. Health professions students' use of social media. J Allied Health. 2011 Summer;40(2):78-81.
38. Adam PC, Murphy DA, de Wit JB. When do online sexual fantasies become reality? The contribution of erotic chatting via the Internet to sexual risk-taking in gay and other men who have sex with men. Health Educ Res. 2011 Jun;26(3):506-15. doi: 10.1093/her/cyq085. Epub 2011 Jan 17.
39. Turner A, Kabashi A, Guthrie H, Burket R, Turner P. Use and value of information sources by parents of child psychiatric patients. Health Info Libr J. 2011 Jun;28(2):101-9. doi: 10.1111/j.1471-1842.2011.00935.x. Epub 2011 Mar 31.
40. Sullivan PS, Khosropour CM, Luisi N, Amsden M, Coggia T, Wingood GM, DiClemente RJ. Bias in online recruitment and retention of racial and ethnic minority men who have sex with men. J Med Internet Res. 2011 May 13;13(2):e38. doi: 10.2196/jmir.1797.
41. Rhodes SD, Hergenrather KC, Vissman AT, Stowers J, Davis AB, Hannah A, Alonzo J, Marsiglia FF. Boys must be men, and men must have sex with women: a qualitative CBPR study to explore sexual risk among African American, Latino, and White gay men and MSM. Am J Mens Health. 2011 Mar;5(2):140-51. doi: 10.1177/1557988310366298. Epub 2010 Apr 21.
42. Friedman DB, Koskan A, Rose ID. Prostate cancer guidelines on Web 2.0-based sites: the screening dilemma continues online. J Cancer Educ. 2011 Mar;26(1):188-93. doi: 10.1007/s13187-010-0180-0.

43. Abraham J, Sick B, Anderson J, Berg A, Dehmer C, Tufano A. Selecting a provider: what factors influence patients' decision making? J Healthc Manag. 2011 Mar-Apr;56(2):99-114; discussion 114-5.
44. Hansen M, Oosthuizen G, Windsor J, Doherty I, Greig S, McHardy K, McCann L. Enhancement of medical interns' levels of clinical skills competence and self-confidence levels via video iPods: pilot randomized controlled trial. J Med Internet Res. 2011 Mar 1;13(1):e29. doi: 10.2196/jmir.1596.
45. Predicting individual affect of health interventions to reduce HPV prevalence. Adv Exp Med Biol. 2011;696:181-90. doi: 10.1007/978-1-4419-7046-6_18.
46. Invited manuscript poster on renal-related education American Society of Nephrology, Nov. 16-21, 2010. Adolescents with chronic kidney disease and their need for online peer mentoring: a qualitative investigation of social support and healthcare transitions. Ren Fail. 2011;33(7):663-8. doi: 10.3109/0886022X.2011.589949.
47. Kishimoto K, Fukushima N. Use of anonymous Web communities and websites by medical consumers in Japan to research drug information. Yakugaku Zasshi. 2011;131(5):685-95.
48. Elkin L, Thomson G, Wilson N. Connecting world youth with tobacco brands: YouTube and the internet policy vacuum on Web 2.0. Tob Control. 2010 Oct;19(5):361-6. doi: 10.1136/tc.2010.035949. Epub 2010 Aug 25.
49. Seeman N, Ing A, Rizo C. Assessing and responding in real time to online anti-vaccine sentiment during a flu pandemic. Healthc Q. 2010 Sep;13 Spec No:8-15.
50. Miller EA, Pole A. Diagnosis blog: checking up on health blogs in the blogosphere. Am J Public Health. 2010 Aug;100(8):1514-9. doi: 10.2105/AJPH.2009.175125. Epub 2010 Jun 17.
51. Mitchell KJ, Finkelhor D, Jones LM, Wolak J. Use of social networking sites in online sex crimes against minors: an examination of national incidence and means of utilization. J Adolesc Health. 2010 Aug;47(2):183-90. doi: 10.1016/j.jadohealth.2010.01.007. Epub 2010 Apr 25.
52. Finn G, Garner J, Sawdon M. 'You're judged all the time!' Students' views on professionalism: a multicentre study. Med Educ. 2010 Aug;44(8):814-25. doi: 10.1111/j.1365-2923.2010.03743.x.
53. Rozental TD, George TM, Chacko AT. Social networking among upper extremity patients. J Hand Surg Am. 2010 May;35(5):819-823.e1. doi: 10.1016/j.jhsa.2009.12.043. Epub 2010 Mar 15.
54. Ahmed OH, Sullivan SJ, Schneiders AG, McCrory P. iSupport: do social networking sites have a role to play in concussion awareness? Disabil Rehabil. 2010;32(22):1877-83. doi: 10.3109/09638281003734409.
55. Kontos EZ, Emmons KM, Puleo E, Viswanath K. Communication inequalities and public health implications of adult social networking site use in the United States. J Health Commun. 2010;15 Suppl 3:216-35. doi: 10.1080/10810730.2010.522689.
56. Shi J, Niu Q. SNSs usage among Chinese internet users: an empirical study. Stud Health Technol Inform. 2010;154:150-4.
57. Chou WY, Hunt YM, Beckjord EB, Moser RP, Hesse BW. Social media use in the United States: implications for health communication. J Med Internet Res. 2009 Nov 27;11(4):e48. doi: 10.2196/jmir.1249.
58. Miyahara M, Butson R, Cutfield R, Clarkson JE. A pilot study of family-focused tele-intervention for children with developmental coordination disorder: development and lessons learned. Telemed J E Health. 2009 Sep;15(7):707-12. doi: 10.1089/tmj.2009.0022.
59. Nordqvist C, Hanberger L, Timpka T, Nordfeldt S. Health professionals' attitudes towards using a Web 2.0 portal for child and adolescent diabetes care: qualitative study. J Med Internet Res. 2009 Apr 6;11(2):e12. doi: 10.2196/jmir.1152.
60. Versteeg KM, Knopf JM, Posluszny S, Vockell AL, Britto MT. Teenagers wanting medical advice: Is MySpace the answer? Arch Pediatr Adolesc Med. 2009 Jan;163(1):91-2. doi: 10.1001/archpediatrics.2008.503. No abstract available.
61. Arsand E, Tufano JT, Ralston JD, Hjortdahl P. Designing mobile dietary management support technologies for people with diabetes. J Telemed Telecare. 2008;14(7):329-32. doi: 10.1258/jtt.2008.007001.

62. Koc M, Gulyagci S. Facebook addiction among Turkish college students: the role of psychological health, demographic, and usage characteristics. Cyberpsychol Behav Soc Netw. 2013 Apr;16(4):279-84. doi: 10.1089/cyber.2012.0249. Epub 2013 Jan 3.
63. Hanberger L, Ludvigsson J, Nordfeldt S. Use of a web 2.0 portal to improve education and communication in young patients with families: randomized controlled trial. J Med Internet Res. 2013 Aug 23;15(8):e175. doi: 10.2196/jmir.2425.
64. Heffner JL, Wyszynski CM, Comstock B, Mercer LD, Bricker J. Overcoming recruitment challenges of web-based interventions for tobacco use: the case of web-based acceptance and commitment therapy for smoking cessation. Addict Behav. 2013 Oct;38(10):2473-6. doi: 10.1016/j.addbeh.2013.05.004. Epub 2013 May 14.
65. Holt M, Rawstorne P, Wilkinson J, Worth H, Bittman M, Kippax S. HIV testing, gay community involvement and internet use: social and behavioural correlates of HIV testing among Australian men who have sex with men. AIDS Behav. 2012 Jan;16(1):13-22. doi: 10.1007/s10461-010-9872-z.
66. Brady SS, Iantaffi A, Galos DL, Rosser BR. Open, closed, or in between: relationship configuration and condom use among men who use the internet to seek sex with men. AIDS Behav. 2013 May;17(4):1499-514. doi: 10.1007/s10461-012-0316-9.
67. Dixon-Gray LA, Mobley A, McFarlane JM, Rosenberg KD. Amor y Salud (Love and Health): a preconception health campaign for second-generation Latinas in Oregon. Am J Health Promot. 2013 Jan-Feb;27(3 Suppl):S74-6. doi: 10.4278/ajhp.120113-ARB-29.
68. Grosskopf NA, Harris JK, Wallace BC, Nanin JE. Online sex-seeking behaviors of men who have sex with men in New York City. Am J Mens Health. 2011 Sep;5(5):378-85. doi: 10.1177/1557988310372801. Epub 2010 Aug 26.
69. Mann TA, Uddin Z, Hendriks AM, Bouchard CJ, Etches VG.Get Tested Why Not? A novel approach to internet-based chlamydia and gonorrhea testing in Canada. Can J Public Health. 2013 Mar 7;104(3):e205-9.
70. Nguyen P, Gold J, Pedrana A, Chang S, Howard S, Ilic O, Hellard M, Stoove M. Sexual health promotion on social networking sites: a process evaluation of The FaceSpace Project. J Adolesc Health. 2013 Jul;53(1):98-104. doi: 10.1016/j.jadohealth.2013.02.007. Epub 2013 Apr 11.
71. Dennison L, Morrison L, Conway G, Yardley L. Opportunities and challenges for smartphone applications in supporting health behavior change: qualitative study. J Med Internet Res. 2013 Apr 18;15(4):e86. doi: 10.2196/jmir.2583.
72. Chan K, Ho S, Lam T. Infodemiology of alcohol use in Hong Kong mentioned on blogs: infoveillance study. J Med Internet Res. 2013 Sep 2;15(9):e192. doi: 10.2196/jmir.2180.
73. Haga SM, Drozd F, Brendryen H, Slinning K. Mamma mia: a feasibility study of a web-based intervention to reduce the risk of postpartum depression and enhance subjective well-being. JMIR Res Protoc. 2013 Aug 12;2(2):e29. doi: 10.2196/resprot.2659.
74. Chunara R, Bouton L, Ayers JW, Brownstein JS. Assessing the online social environment for surveillance of obesity prevalence. PLoS One. 2013 Apr 24;8(4):e61373. doi: 10.1371/journal.pone.0061373. Print 2013.
75. Gilroy H, McFarlane J, Nava A, Maddoux J. Preferred communication methods of abused women. Public Health Nurs. 2013 Sep-Oct;30(5):402-8. doi: 10.1111/phn.12030. Epub 2013 Feb 14.
76. Berntsson LT, Ringsberg KC. Health and relationships with leisure time activities in Swedish children aged 2-17¬†years. Scand J Caring Sci. 2013 Sep 30. doi: 10.1111/scs.12081. [Epub ahead of print]
77. Radin P. To me, it's my life": medical communication, trust and activism in cyberspace." Soc Sci Med. 2006 Feb;62(3):591-601. Epub 2005 Jul 20.
78. Berntsen E, Babic A. Cherry: mobile application for children with cancer. Stud Health Technol Inform. 2013;192:1168.
79. Kutz D, Shankar K, Connelly K. Making sense of mobile- and web-based wellness information technology: cross-generational study. J Med Internet Res. 2013 May 14;15(6):e83. doi: 10.2196/jmir.2124.

80. Chen W, Lee KH. Sharing, liking, commenting, and distressed? The pathway between Facebook interaction and psychological distress. Cyberpsychol Behav Soc Netw. 2013 Oct;16(10):728-34. doi: 10.1089/cyber.2012.0272. Epub 2013 Jun 7.

81. Hildebrand M, Ahumada C, Watson S. CrowdOutAIDS: crowdsourcing youth perspectives for action. Reprod Health Matters. 2013 May;21(41):57-68. doi: 10.1016/S0968-8080(13)41687-7.

82. Mitter N, Nah GQ, Bong YL, Lee J, Chong SA. Longitudinal Youth-At-Risk Study (LYRIKS): outreach strategies based on a community-engaged framework. Early Interv Psychiatry. 2013 May 20. doi: 10.1111/eip.12049. [Epub ahead of print]

83. Hanson CL, Burton SH, Giraud-Carrier C, West JH, Barnes MD, Hansen B. Tweaking and tweeting: exploring Twitter for nonmedical use of a psychostimulant drug (Adderall) among college students. J Med Internet Res. 2013 Apr 17;15(4):e62. doi: 10.2196/jmir.2503.

84. Morgan AJ, Jorm AF, Mackinnon AJ. Internet-based recruitment to a depression prevention intervention: lessons from the Mood Memos study. J Med Internet Res. 2013 Feb 12;15(2):e31. doi: 10.2196/jmir.2262.

85. Balfe M, Doyle F, Conroy R. Using Facebook to recruit young adults for qualitative research projects: how difficult is it? Comput Inform Nurs. 2012 Oct;30(10):511-5. doi: 10.1097/NXN.0b013e31826e4fca. No abstract available.

86. D'Alessandro AM, Peltier JW, Dahl AJ. A large-scale qualitative study of the potential use of social media by university students to increase awareness and support for organ donation. Prog Transplant. 2012 Jun;22(2):183-91. doi: 10.7182/pit2012619.

87. Bramlett Mayer A, Harrison JA. Safe Eats: an evaluation of the use of social media for food safety education. J Food Prot. 2012 Aug;75(8):1453-63. doi: 10.4315/0362-028X.11-551.

88. Shenouda C, Hendrickson P, Davenport K, Barber J, Bell KR. The effects of concussion legislation one year later--what have we learned: a descriptive pilot survey of youth soccer player associates. PM R. 2012 Jun;4(6):427-35. doi: 10.1016/j.pmrj.2012.02.016. Epub 2012 Apr 28.

89. Nan X, Madden K. HPV vaccine information in the blogosphere: how positive and negative blogs influence vaccine-related risk perceptions, attitudes, and behavioral intentions. Health Commun. 2012 Nov;27(8):829-36. doi: 10.1080/10410236.2012.661348. Epub 2012 Mar 27.

90. Bender JL, Wiljer D, To MJ, Bedard PL, Chung P, Jewett MA, Matthew A, Moore M, Warde P, Gospodarowicz M. Testicular cancer survivors' supportive care needs and use of online support: a cross-sectional survey. Support Care Cancer. 2012 Nov;20(11):2737-46. doi: 10.1007/s00520-012-1395-x. Epub 2012 Mar 3.

91. Gajaria A, Yeung E, Goodale T, Charach A. Beliefs about attention-deficit/hyperactivity disorder and response to stereotypes: youth postings in Facebook groups. J Adolesc Health. 2011 Jul;49(1):15-20. doi: 10.1016/j.jadohealth.2010.09.004. Epub 2011 Apr 2.

92. Sullivan SJ, Schneiders AG, Cheang CW, Kitto E, Lee H, Redhead J, Ward S, Ahmed OH, McCrory PR. 'What's happening?' A content analysis of concussion-related traffic on Twitter. Br J Sports Med. 2012 Mar;46(4):258-63. doi: 10.1136/bjsm.2010.080341. Epub 2011 Mar 15.

93. Moreno MA, Jelenchick LA, Egan KG, Cox E, Young H, Gannon KE, Becker T. Feeling bad on Facebook: depression disclosures by college students on a social networking site. Depress Anxiety. 2011 Jun;28(6):447-55. doi: 10.1002/da.20805. Epub 2011 Mar 11.

94. Bender JL, Jimenez-Marroquin MC, Jadad AR. Seeking support on facebook: a content analysis of breast cancer groups. J Med Internet Res. 2011 Feb 4;13(1):e16. doi: 10.2196/jmir.1560.

95. Tian Y. Organ donation on Web 2.0: content and audience analysis of organ donation videos on YouTube. Health Commun. 2010 Apr;25(3):238-46. doi: 10.1080/10410231003698911.

96. Rogers VL, Griffin MQ, Wykle ML, Fitzpatrick JJ. Internet versus face-to-face therapy: emotional self-disclosure issues for young adults. Issues Ment Health Nurs. 2009 Oct;30(10):596-602. doi: 10.1080/01612840903003520.

97. Mallette C, Duff M, McPhee C, Pollex H, Wood A. Workbooks to virtual worlds: a pilot study comparing educational tools to foster a culture of safety and respect in Ontario. Nurs Leadersh (Tor Ont). 2011 Jan;24(4):44-64.
98. Reavley NJ, Mackinnon AJ, Morgan AJ, Alvarez-Jimenez M, Hetrick SE, Killackey E, Nelson B, Purcell R, Yap MB, Jorm AF. Quality of information sources about mental disorders: a comparison of Wikipedia with centrally controlled web and printed sources. Psychol Med. 2012 Aug;42(8):1753-62. doi: 10.1017/S003329171100287X. Epub 2011 Dec 14.
99. Kamel Boulos MN, Toth-Cohen S. The University of Plymouth Sexual Health SIM experience in Second Life: evaluation and reflections after 1 year. Health Info Libr J. 2009 Dec;26(4):279-88. doi: 10.1111/j.1471-1842.2008.00831.x.
100. Wicks P, Keininger DL, Massagli MP, de la Loge C, Brownstein C, Isojrvi J, Heywood J. Perceived benefits of sharing health data between people with epilepsy on an online platform. Epilepsy Behav. 2012

Papers not clearly involving Online Social Media (n=30)

1. Mycoplasma pneumoniae outbreak at a university - Georgia, 2012. MMWR Morb Mortal Wkly Rep. 2013 Aug 2;62(30):603-6.
2. Imdad A, Tserenpuntsag B, Blog DS, Halsey NA, Easton DE, Shaw J. Religious exemptions for immunization and risk of pertussis in New York State, 2000-2011. Pediatrics. 2013 Jul;132(1):37-43. doi: 10.1542/peds.2012-3449. Epub 2013 Jun
3. Muessig KE, Pike EC, Fowler B, LeGrand S, Parsons JT, Bull SS, Wilson PA, Wohl DA, Hightow-Weidman LB. Putting prevention in their pockets: developing mobile phone-based HIV interventions for black men who have sex with men. AIDS Patient Care STDS. 2013 Apr;27(4):211-22. doi: 10.1089/apc.2012.0404.
4. Weller JA, Shackleford C, Dieckmann N, Slovic P. Possession attachment predicts cell phone use while driving. Health Psychol. 2013 Apr;32(4):379-87. doi: 10.1037/a0029265. Epub 2012 Aug 27.
5. Woreta SA, Kebede Y, Zegeye DT. Knowledge and utilization of information communication technology (ICT) among health science students at the University of Gondar, North Western Ethiopia. BMC Med Inform Decis Mak. 2013 Mar 3;13:31. doi: 10.1186/1472-6947-13-31.
6. Herbert DL, Loxton D, Bateson D, Weisberg E, Lucke JC. Challenges for researchers investigating contraceptive use and pregnancy intentions of young women living in urban and rural areas of Australia: face-to-face discussions to increase participation in a web-based survey. J Med Internet Res. 2013 Jan 21;15(1):e10. doi: 10.2196/jmir.2266.
7. Applebaum MA, Lawson EF, von Scheven E. Perception of transition readiness and preferences for use of technology in transition programs: teens' ideas for the future. Int J Adolesc Med Health. 2013;25(2):119-25. doi: 10.1515/ijamh-2013-0019.
8. Chi X, Yu L, Winter S. Prevalence and correlates of sexual behaviors among university students: a study in Hefei, China. BMC Public Health. 2012 Nov 13;12:972. doi: 10.1186/1471-2458-12-972.
9. Park N, Lee H. Social implications of smartphone use: Korean college students' smartphone use and psychological well-being. Cyberpsychol Behav Soc Netw. 2012 Sep;15(9):491-7. doi: 10.1089/cyber.2011.0580. Epub 2012 Jul 20.
10. Beaulieu D, Godin G. Development of an intervention programme to encourage high school students to stay in school for lunch instead of eating at nearby fast-food restaurants. Eval Program Plann. 2012 Aug;35(3):382-9. doi: 10.1016/j.evalprogplan.2012.01.004. Epub 2012 Jan 16.
11. Mazurek MO, Shattuck PT, Wagner M, Cooper BP. Prevalence and correlates of screen-based media use among youths with autism spectrum disorders. J Autism Dev Disord. 2012 Aug;42(8):1757-67. doi: 10.1007/s10803-011-1413-8.

12. Akinfaderin-Agarau F, Chirtau M, Ekponimo S, Power S. Opportunities and limitations for using new media and mobile phones to expand access to sexual and reproductive health information and services for adolescent girls and young women in six Nigerian states. Afr J Reprod Health. 2012 Jun;16(2):219-30.
13. Protudjer JL, McGavock JM, Ramsey CD, Sevenhuysen GP, Kozyrskyj AL, Becker AB. "Asthma isn't an excuse, it's just a condition": youths' perceptions of physical activity and screen time. J Asthma. 2012 Jun;49(5):496-501. doi: 10.3109/02770903.2012.680637. Epub 2012 May 3.
14. Shoveller J, Knight R, Davis W, Gilbert M, Ogilvie G. Online sexual health services: examining youth's perspectives. Can J Public Health. 2012 Jan-Feb;103(1):14-8.
15. Gold J, Lim MS, Hellard ME, Hocking JS, Keogh L. What's in a message? Delivering sexual health promotion to young people in Australia via text messaging. BMC Public Health. 2010 Dec 29;10:792. doi: 10.1186/1471-2458-10-792.
16. Draucker CB, Martsolf DS. The role of electronic communication technology in adolescent dating violence. J Child Adolesc Psychiatr Nurs. 2010 Aug;23(3):133-42. doi: 10.1111/j.1744-6171.2010.00235.x.
17. Torsheim T, Eriksson L, Schnohr CW, Hansen F, Bjarnason T, Välimaa R. Screen-based activities and physical complaints among adolescents from the Nordic countries. BMC Public Health. 2010 Jun 9;10:324. doi: 10.1186/1471-2458-10-324.
18. Crosby RA, Yarber WL, Graham CA, Sanders SA. Does it fit okay? Problems with condom use as a function of self-reported poor fit. Sex Transm Infect. 2010 Feb;86(1):36-8. doi: 10.1136/sti.2009.036665.
19. Whittaker R, Maddison R, McRobbie H, Bullen C, Denny S, Dorey E, Ellis-Pegler M, van Rooyen J, Rodgers A. A multimedia mobile phone-based youth smoking cessation intervention: findings from content development and piloting studies. J Med Internet Res. 2008 Nov 25;10(5):e49. doi: 10.2196/jmir.1007.
20. Schaffzin JK, Pollock L, Schulte C, Henry K, Dayan G, Blog D, Smith P. Effectiveness of previous mumps vaccination during a summer camp outbreak. Pediatrics. 2007 Oct;120(4):e862-8.
21. Kim YR, Son JW, Lee SI, Shin CJ, Kim SK, Ju G, Choi WH, Oh JH, Lee S, Jo S, Ha TH. Abnormal brain activation of adolescent internet addict in a ball-throwing animation task: possible neural correlates of disembodiment revealed by fMRI. Prog Neuropsychopharmacol Biol Psychiatry. 2012 Oct 1;39(1):88-95. doi: 10.1016/j.pnpbp.2012.05.013. Epub 2012 Jun 9.
22. Kang M, Rochford A, Skinner R, Mindel A, Webb M, Peat J, Usherwood T. Facilitating chlamydia testing among young people: a randomised controlled trial in cyberspace. Sex Transm Infect. 2012 Dec;88(8):568-73. doi: 10.1136/sextrans-2011-050124. Epub 2012 Jul 4.
23. Voisin D, Shiu CS, Chan Tack A, Krieger C, Sekulska D, Johnson L. In their own words: racial/ethnic and gender differences in sources and preferences for HIV prevention information among young adults. AIDS Care. 2013;25(11):1407-10. doi: 10.1080/09540121.2013.772274. Epub 2013 Mar 1.
24. Akinyanju OO, Otaigbe AI, Ibidapo MO. Outcome of holistic care in Nigerian patients with sickle cell anaemia. Clin Lab Haematol. 2005 Jun;27(3):195-9.
25. Sheese BE, Brown EL, Graziano WG. Emotional expression in cyberspace: searching for moderators of the Pennebaker disclosure effect via e-mail. Health Psychol. 2004 Sep;23(5):457-64. Erratum in: Health Psychol. 2006 Jul;25(4):iii.
26. Larner AJ. Use of internet medical websites and NHS direct by neurology outpatients before consultation. Int J Clin Pract. 2002 Apr;56(3):219-21.
27. Hallett J, Brown G, Maycock B, Langdon P. Changing communities, changing spaces: the challenges of health promotion outreach in cyberspace. Promot Educ. 2007;14(3):150-4.
28. Labacher L, Mitchell C. Talk or Text to Tell? How Young Adults in Canada and South Africa Prefer to Receive STI Results, Counseling, and Treatment Updates in a Wireless World. J Health Commun. 2013 Dec;18(12):1465-76. doi: 10.1080/10810730.2013.798379. Epub 2013 Sep 9.
29. Fogel J, Krausz F. Watching reality television beauty shows is associated with tanning lamp use and outdoor tanning among college students. J Am Acad Dermatol. 2013 May;68(5):784-9. doi: 10.1016/j.jaad.2012.09.055. Epub 2012 Dec 21.

30. Dhar M, Lahiri S, Takiar R, Ashok NC, Murthy NS. An indirect study of cancer survival in the context of developing countries. Asian Pac J Cancer Prev. 2008 Jul-Sep;9(3):479-86.

Papers not pertaining to Health (n=1)

1. Martinello N, Donelle L. Online conversations among Ontario university students: environmental concerns. Inform Health Soc Care. 2012 Sep;37(3):177-89. doi: 10.3109/17538157.2012.678448. Epub 2012 Jun 19.
