# Supplementary material for: “Friending” Teens: Systematic Review of Social Media in Adolescent and Young Adult Health Care
Source: J Med Internet Res. 2015 Jan 5;17(1):e4. doi: 10.2196/jmir.3692 (PMC4376201; doi:10.2196/jmir.3692)
Supplement: Supplementary file 2 [file medinform_v17i1e297_app2.pdf]

Multimedia Appendix 2: Summary table of studies included in systematic review.

Authors	Purpose	Social Media Used	Participants/ Sample Size	Methods	Significant Positive / Negative Findings
Tichon & Shapiro (2003)	To explore the supportive messages that are produced and responded to in an online support group for children who have a sibling with special needs	Sibkids Email listserv	655 separate emails from children who have a sibling with special needs over 3 months originating from 58 unique senders	Qualitative and quantitative content analysis of the Sibkids listserv postings for types of social support (informational, emotional, social companionship) exchanged, and the nature of self-disclosure shared by individual participants, use of descriptive statistics to capture important themes within the data	Self-disclosure was used in 3 different ways: 1) it functioned for the support recipient to initiate a transactional relationship with another member for the purpose of attracting social support through the open expression of concerns and frustrations; 2) it is then used by the support provider to demonstrate that coping is possible for the recipient through the reciprocal self-disclosure of similar concerns and situations with which the member has successfully dealt; 3) to share reciprocal social companionship relationships.
Ybarra et al. (2006)	To identify the characteristics of youth who are targets of Internet harassment & characteristics related to reporting distress as a result of the incident	SNS	1500 individuals 10-17 years old & their primary adult caregiver	Telephone questionnaire regarding internet use and harassment	The majority have not been harassed, but among those who are, the majority are not impacted emotionally by the event & disclose it to a peer or adult; though a minority, 1 in 3 targets of Internet harassment report being harassed 3 or more times, either by the same person or different people; social problems & online aggressive behavior are each associated with elevated odds of being the target of harassment

Pantic et al. (2012)	To investigate the relationship between social networking and depression indicators in adolescent population	SNS including mention of Facebook	160 high school students, avg. age 18 years old	Interviews using an anonymous, structured questionnaire and Back Depression Inventory - 2nd edition (BDI-II-II)	The time spent on SNS was found to be related to the risk for depression determined by BDI-II
O'Dea & Campbell (2011)	To examine the use of SNS in a rural town, and the potential of SNS to be used for preventative mental health care	SNS	74 high-school students in a rural town in Australia (mean age 14.7 years old)	Paper questionnaire regarding internet, SNS use & perceptions of mental health support	Participants who use SNS are less likely to engage anonymously elsewhere (such as chat sites); over 50% had gone online to find help on a mental health concern; participants felt least comfortable approaching formal sources of help (doctors, counselors, etc.)
Moreno et al. (2007)	To examine 16- and 17-year-old Myspace profiles and determine the prevalence of personal risk behavior descriptions and identifiable information	Myspace	142 profiles of individuals 16-17years old	Content analysis of Myspace profiles	Most profiles (over 70%) included identifiable information, are frequently accessed by owners, and almost half (47%) included disclosure of sexual activity or substance use
Moreno et al. (2010a)	To assess displayed alcohol references on Myspace profiles	Myspace	400 profiles created by individuals within one county in Washington state, 17-20 years old	Content analysis of Myspace profiles	Despite alcohol use being illegal and potentially stigmatizing in this population, explicit alcohol use is frequently referenced on adolescents' Myspace profiles across several sociodemographic communities
Egan & Moreno (2011a)	To perform a content analysis of Facebook profiles for references to	Facebook Profiles	225 college males, 18-23 years old	Content analysis of public Facebook profiles using a previously used SNS codebook	Students who were of legal drinking age referenced alcohol 4.5 times more than underage students; and increase

	alcohol use including timing and content of alcohol references				reference was seen when entering college, and an increase in number of Facebook friends was associated with an increase in displayed alcohol references
Griffiths & Casswell (2010)	To examine how young people in New Zealand engage with alcohol and reproduce alcohol marketing messages & alcohol-related branding in Bebo	Bebo	150 profiles created by individuals 16-20 years old	Ethnographic content analysis of personal & fan Bebo profiles	Young people are creating 'intoxigenic social identities' as well as 'intoxigenic digital spaces' that further contribute towards the normalization of youth consumption of alcohol
Ridout et al. (2012)	To investigate identity construction through autophotography (the photographic essay) to provide insight into the portrayal of 'alcohol-identity' on Facebook	Facebook	158 college students, 17-24 years old	Descriptive analysis of all photos linked to a participants' Facebook profile after friending a research profile	Portraying oneself as a drinker is considered by many young people to be a socially desirable component of identity in the SNS environment, perpetuating an online culture that normalizes binge drinking; 60% of college participants reported potentially problematic alcohol use according to Alcohol Use Disorders identification
Whitehill et al. (2013)	To determine acceptability, preferred communication medium, etc. for intervention by friends/professors/resident advisors/strangers in response to college students' references to depression on	Facebook Profiles	60 1st-year college students (60% female), 18-19 years old from one large state university	Content analysis of public Facebook profiles for depression references and 1:1 interviews with participants regarding acceptibility and preferred method of communication about depression	Face-to-face communication from friends & trusted adults is the preferred means for raising concerns about a student's signs of depression displays on Facebook; participants indicated a preference for more direct forms of communication as opposed to indirect/electronic means; participants,

	Facebook				especially those who displayed signs of depression on Facebook, did not approve of individuals with whom they do not have an offline relationship reaching out to them about the topic
Jenssen et al. (2009)	To describe exposure to tobacco and smoking-related text & images on webpages and Myspace pages	SNS	346 individuals, 14-17 years old	Content analysis of webpages and Myspace pages collected from tracking of participant online activity using software, online questionnaire	Most tobacco references were found on SNS, and Myspace sites alone represented almost half of all pages containing tobacco content; overall, only a small proportion of websites visited by participants contained tobacco messages
Pujazon-Zazik et al. (2012)	To analyze teen profiles on a teen dating website for health risk content	myLol.net, a teen dating website	752 individuals, 14-18 years old	Content analysis of public profiles	Fewer adolescents include risky content vs. previous data from Myspace profiles; females were more likely to include mention of interest/participation in sexual risk behaviors possibly as a result of internalized social norms that place a high value on female sexuality; no significant age group differences
Moreno et al. (2010b)	To evaluate whether online friends of adolescents who display sexual references on a SNS also display references	Myspace	180 individuals & friends, 18 years old	Content analysis of Myspace profiles: 20 initial + 160 ("top 8" friends)	Participants who displayed explicit sexual references were more likely to have online friends who displayed references in a similar pattern
Moreno et al. (2011)	To assess male college students views & expectations about sexual references displayed on	SNS	28 college males from one large state university, 18-23 years old	Descriptive analysis of 7 small focus group discussions	Females who display sexual references on publicly available SNS profiles may be influencing potential partners' sexual expectations & dating

	SNS by females				intentions
Dunlop et al. (2011)	To determine whether Internet sites, such as online news & SNS, expose young people to suicide stories that might increase suicide ideation	SNS	719 individuals, 14-24 years old	Phone interview with follow-up interview/online questionnaire 1 year later	The Internet & especially SNS are important sources of suicide stories; youth who had experienced hopelessness and those who were heavier SNS users and the internet were more likely to engage in blogging
Ybarra & Mitchell (2008)	To describe the frequency of unwanted sexual solicitation online, including on SNS	SNS	1588 individuals, 10-15 years old & their primary adult caregiver	Online questionnaires	The majority who are targeted do not report it occurring on a SNS: 15 % of all youth reported being targeted by unwanted sexual solicitation online, with 4% specifically in a SNS; 32.5% report being harassed (threats, aggressive comments, rumors), with 9% specifically on a SNS
Wang et al. (2011)	To examine the association between depression & frequency of involvement in physical, verbal, relational and cyber forms of bullying and to compare levels of depression among bullies, victims, and bully-victims	SNS	7313 individuals in grades 6 to 10 (mean age 14.2)	Anonymous self-reported paper questionnaire	Cyber victims reported higher depression than bullies or bully-victims, which was not found in any other form of bullying

Marcus et al. (2012)	To investigate the blogs of young adults with mental health concerns so as to better understand their experiences	Blogs	8 blogs created by those 18-25 years old	Grounded theory & qualitative content analysis of blogs	Bloggers expressed significant feelings of powerlessness as a result of their mental health concerns and simultaneously felt a profound sense of loneliness, alienation, and lack of connection with others; half of bloggers stated that they used blogging as a means of coping, and found it that helped them through self-reflection, self-help, and acquiring much-needed support
Lam et al. (2013)	To assess the quality, readability & social reach of websites on osteosarcoma for adolescents	Websites with links to social media or SNS	56 websites	Social reach was gauged by SNS links, global website traffic & a pilot adolescent-specificity measure	Most websites offered at least one form of social media, and one-third of websites utilized at least one site-specific form of social media (such as an interactive blog, community page, or public SNS) to engage users
Clerici et al. (2012)	To describe the availability & type of video content and origin on YouTube relating to a particular set of pediatric neoplastic diseases (rhabdomyosarcoma & soft-tissue sarcoma)	YouTube	149 YouTube videos	Qualitative analysis of videos	Majority of videos were amateur productions by patients or family members; there were more videos commemorating those who died than about cases that were cured
Nordfeldt et al. (2010)	To explore patients' and parents' attitudes toward a Web 2.0 portal for patients with type-1 diabetes (DM)	Web 2.0 portal website "Diabit"	16 mothers & 3 fathers of patients with type-1 DM, 5 patients with type-1 DM	Qualitative analysis of essay responses to questions posed about the portal	Web 2.0 services have great potential for supporting parents and patients with type 1 diabetes by enhancing their information retrieval & disease management; content should be carefully maintained and

					updated by health care professionals who are alert and active on the site; the support from peers is of added value; login procedures should be simple and minimized as much as possible; the education of clinical practitioners regarding the use of Web 2.0 resources needs more attention
D'Alessa ndroet al. (2012)	To investigate how college students can be social support catalysts for organ donation and how social, cognitive & attitudinal dimensions impact organ donor registration	Social media campaigns on Facebook, Twitter, YouTube, and project blog	317 college-students in exploratory phase, 1800 responses to subsequent questionnaire; all members of Collegiate American Marketing Association	Exploratory interviews & focus groups which informed the creation of online questionnaires	Within the student organization, organ donor registration increased by 28%; 18-month social media campaign collected 4473 Facebook members, 1189 YouTube video submissions with 164,000 views, motivated 19,623 people to go to a state's organ donor registration page, and had 9000 documented organ donor registrations
Divecha et al. (2012)	To investigate how low-income parenting adolescents & young adults communicate about sexual health with peers using new media technologies	"New media technologies" (SNS)	94 parenting adolescents/y oung adults, 14-21 years old	Structured audio & computer-assisted self-interviews about use of new media technologies, communication with friends about sexual health and willingness to use new media technologies for communication	For conversations about sexual health, young urban parents prefer private forms of communication; race was associated with willingness: African Americans made up largest proportion that was willing, Latinos the largest proportion that were unwilling; those willing to communicate has greater condom self-efficacy, greater STD knowledge, and more Facebook friends

Moreno et al. (2009a)	To determine whether an online email intervention reduces references to sex & substance abuse on Myspace among at-risk adolescents	Myspace	190 individuals 18-20 years old deemed "at-risk"	Randomized controlled Pilot Intervention Trial; Content analysis of Myspace profiles before & 3 mths after email intervention from researchers posing as an adolescent medicine physician, "Dr. Meg"	Brief email interventions through SNS show promise in reducing sexual references in online profiles of at-risk adolescents; females may be especially more open to feedback regarding their Web profiles& subsequently alter online behaviors
Hedge & Donald (2011)	To explore the effectiveness and specific challenges of the Myspace project, which was develop to gain feedback on how to improve services and make sexual health information accessible to a larger and hard to reach audience	Myspace, Facebook, Bebo, High5	78 individuals, 15-25 years old	Created group study profile on each SNS, questionnaires completed by participants who viewed profile(s)	Little engagement on the profile site, possibly due to a reluctance to be identified as a service user
Ramo et al. (2012)	To examine Facebook as a mechanism to reach and survey young adults about tobacco and other substance use	Facebook Ads for recruitment	1548 cigarette users 18-25 years old	Recruitment for online questionnaire about tobacco and other substance use using targeted Facebook Ads	Despite wide variety in the success of individual ads & potential concerns about sample representativeness, Facebook was a useful & cost-effective recruitment source; nearly half (48%) reported being unwilling to quit smoking within the next 6 months; Facebook guidelines limit use of language/pictures related to illegal activity and thereby widen target subject pool

Moreno et al. (2012b)	To determine associations between displayed depression symptoms on Facebook and self-reported depression via a depression screening questionnaire	Facebook Profiles	215 college students from two large state universities, 18-20 years old	Content analysis of public Facebook profiles using a previously used SNS codebook, online questionnaire to evaluate depressive symptoms	Displayed depression symptom references on Facebook were positively associated with PHQ-9 scores; despite the potential for stigma, approximately 33% of participants displayed depression symptom references on their Facebook profiles
Moreno et al. (2012c)	To assess older adolescents' responses after learning that they were participants in a research study that involved identification of participants using Facebook	Facebook Profiles	132 1st-year college students from one large state university, 18-19 years old	Qualitative descriptive analysis of semi-structured interviews about being identified on Facebook for a research study	The majority of adolescent participants viewed the use of Facebook for research positively; those who expressed concerns also voiced confusion regarding their current profile security settings (possible that these reactions were rooted in concerns regarding these participants' understanding of profile security settings)
Moreno et al. (2012a)	To investigate college students' views towards potential SNS screening for intervention efforts regarding alcohol	Facebook	132 1st-year college students, 18-19 years old	Qualitative analysis of interviews	Participants reported that they would prefer to be approached in a direct manner by someone they knew to address worrisome alcohol references, but would also be open to approach by resident advisors or professors; and the style of approach was considered critical (open but direct, with good intention); use of Facebook as a medium of discussion may be helpful

Bauermeister et al. (2012)	To examine whether web-based respondent-driven sampling (webRDS) could be used to recruit young adults into a web questionnaire designed to assess alcohol and other drug (AOD) use	Facebook Ads and webRDS for recruitment	22 "seeds" recruited via Facebook with remainder 3,426 recruited via webRDS method, 18-24 years old	Recruitment of 22 webRDS seeds via targeted Facebook Ads, followed by dissemination through referrals	webRDS helped overcome physical & temporal barriers that helped expedite study recruitment; the asynchronous nature of online communication served as an added recruitment advantage because participants could invite peers even if they weren't online at the same time
Stoddard et al. (2012)	To examine the association between the presence of alcohol and other drug (AOD) use content in online social networking and perceived norms (online norms regarding AOD use & anticipated regret with AOD use postings)	SNS, Facebook Ads and webRDS for recruitment	3448 individuals, 18-24 years old	Online questionnaire regarding AOD use, norms & peer support	Those who reported a greater prevalence of alcohol content (e.g., pictures, wall posts) in their online profiles were also more likely to report greater alcohol use; peers play an important role in young adult AOD use, whether online or in person; young adults who had greater emotional support from online friends were less likely to report recent alcohol use; once prevalence of alcohol content was accounted for, there was no relationship between promotive AOD norms online & alcohol use
Jones L et al. (2012)	To describe the use of the social networking website in the re-recruitment & tracking of adolescent girls into a follow-up study	Facebook Profile created for study purposes	175 adolescent girls in 11th grade who were lost to follow-up for initial study with 730 girls	Facebook study profile established to search for & contact participants who couldn't be reached by traditional recruitment methods	Loss to follow-up was minimized by contacting through Facebook: ultimately re-recruited 6% of participants who would have otherwise been lost to follow-up; Facebook was cost-effective (free) compared to traditional methods (snail mail)

Fenner et al. (2012)	To assess the feasibility of recruiting young females for a research study using targeted advertising on Facebook	Facebook Ads for recruitment	278 females, 16-25 years old	Recruitment for online or in-person questionnaire using targeted Facebook Ads	Recruitment yielded a demographically representative sample and was cost-effective; it allowed for flexibility to monitor real-time performance of recruitment efforts; the ability to reach those in rural areas and overweight/obese individuals indicate a potential utility in recruiting those traditionally underrepresented in research, high-risk individuals, and those with specific health conditions
Selkie et al. (2011)	To determine adolescents' views regarding how new technologies (SNS and SMS) could be used for sexual health education and identify themes that would assist providers and educators who may be interested in providing electronic sexual health ed.	SNS	29 individuals (65.5% female, 62.1% heterosexual), 14-19 years old	Small focus groups were designed to explore ways AYAs use SNS and SMS and their views regarding using these technologies for sexual health information; transcripts were analyzed using the constant comparative method for common themes	Themes common to the resources adolescents currently use/would like to use for sexual health information: accessibility, trustworthiness, credibility & confidentiality, personal comfort; participants expressed that they used resources like Google to find information because they felt this was the only method available and that they would rather get answers via personal communication with a credible source, but would like it to be anonymous
Moreno et al. (200b)	To determine the prevalence of/associations among displayed health risk behavior information in sample of	Myspace	500 individuals, 18 years old	Content analysis of Myspace profiles	Just over half (54%) of profiles referenced 1 or more health risk behaviors (substance use, sexual activity, violence)

	adolescents' Myspace profiles				
Gao et al. (2013)	To examine the manifestations, impact, and origins of dental fear & anxiety (DFA) in children & adolescents from the public's perspective using YouTube	YouTube	27 videos involving children/adolescents associated with keywords relating to DFA	Thematic content analysis of YouTube videos	About half of the videos had been viewed hundreds to thousands of times (5 were very popular, with more than 10,000 views each); videos revealed multifaceted manifestations, impacts, and origins of DFA; the nature and extent of psychological impacts were striking (especially internalization of fear, which may lead to psychological or behavioral withdrawal, feelings of shame or inferiority & low self-esteem)
Egan & Moreno (2011b)	To evaluate whether Facebook profiles might be useful for screening students at risk for stress-related conditions	Facebook profiles	300 college students from one large state university, 18-19 years old	Content analysis of public Facebook profiles using a previously used SNS codebook	Stress references were present on 46% of the female profiles, and the majority of stress references were made by females (78%); stress references were most commonly displayed in status updates (98%); positive association between referencing stress and both weight concerns & depressive symptoms
Stokes (2007)	To investigate sexual scripts, self-definition (identity) and hip hop culture in home pages (of an SNS site) constructed by Black girls aged 14-17 living in southern USA	NevaEvaLand, an SNS for Black adolescents	Core sample of 216 pages, 27 pages for in-depth analysis; females 14-17 years old who self-identified as Black, African-American, or of African descent	Ethnographic and quantitative content analysis of pages, verification of preliminary themes/conclusion via consultation with "expert panel" of young Black women	An association was found between sexual scripts and hip-hop music; sexuality education programmes that ignore the role of media in the lives of Black girls may be ineffective; media messages and girls' self-representations in their pages reflect

			residing in 1 of 7 southern states		racism, misogyny, patriarchy, and capitalism in American culture
Villard & Moreno (2012)	Determine a) how and to what extent college students are discussing fitness on Facebook and b) how user-generated fitness information is linked to advertisements for fitness products and advice.	Facebook Profiles and Ads	1) 60 Facebook profiles of college freshmen 18-20 years old within a university network on Facebook, 2) 800 Facebook Ads	Content analysis of Facebook profiles for displayed fitness references and content analysis of Facebook ads elicited by fitness-related Facebook posts	About 72% of profiles referenced one or more fitness behaviors; 97.6% referenced exercise, 4.9% dieting, and 4.9% unhealthy eating. Among the first 40 ads linked to generated status updates, 40.3% were fitness related but not very relevant or accessible to the population: most ads were for charity runs (30.4%), fitness apparel (24.2%), or fad diets (9.9%)
Cash et al. (2013)	To explore the ways in which adolescents use Myspace to comment on their suicidal thoughts and intentions through content analysis of Myspace profiles	Myspace Profiles	64 relevant comments from public Myspace profiles of individuals 13-24 years old	Content analysis of comments extracted from profiles that reflected potential suicidality	Existence of serious suicidal comments on Myspace pages, indicating that adolescents may reach out using SNS for help & support; the majority of comments had no context but 42% pertained to relationships including: break-ups, family relationships, and friendships; 6.3% of statements involved references to mental health problems

Lefkowitz et al. (2012)	To examine a student-constructed holiday, "State Patty's Day," at a university with a dominant drinking culture and the use of Facebook in the spread of information regarding the event, the effects of the holiday on individuals' drinking behaviors, and community-level crime on the holiday.	Facebook groups	11 Facebook groups that referred to the 2007 holiday, 227 1st year college students at a large state university, 18-20 years old (51% male)	Content analysis of Facebook groups and online questionnaire sent via emails to students	Messages about the holiday on Facebook focused on drinking & social aspects of the holiday including the social context of drinking, a sense of belonging to a larger community, and the social norms of drinking; messages were rarely about consequences and were rarely negative in nature
Robertson et al. (2012)	To describe an adolescent suicide cluster that drew attention to the possible role of online social networking and SMS as sources of contagion after a suicide and obstacles to recognition of a potential cluster	Bebo	Individuals from a rural city district in New Zealand, 15-18 years old	Public health approach to analysis of Bebo profiles, interviews and multidisciplinary community response, probability of a time-space cluster using Monte Carlo simulation	Evidence from analysis provided support for a cluster of six deaths in a 6-month period; several suicides were linked by SNS, including sites created in memory of earlier suicide cases; mobile phones also facilitated the rapid spread of information and rumor about the deaths throughout the community, which made the recognition and management of a possible cluster more difficult

Brockman et al. (2012)	1) confirm prevalence of hookah use among US college students 2) identify substances commonly smoked in hookahs and other substance use characteristics of hookah smokers 3) to assess the prevalence of hookah references on Facebook profiles	Facebook	307 profiles of college students within 2 university networks on Facebook, 18-20 years old, 216 of which also completed a questionnaire	Questionnaire evaluating hookah use was administered to those who had their public profiles analyzed for hookah references	Hookah references were present on 5% of Facebook profiles; response rate for the questionnaire was 70%
O'Dea & Campbell (2012)	To explore perceptions & frequency of cyber-bullying experiences and to examine the relationship between SNS use and the experience of cyber-bullying	SNS	400 Australian high-school students, 12-17 years old	Online self-reported questionnaire	51.5% of participants reported that they accept friend requests from unknown people on SNS; ownership of a SNS profile may be a stronger predictor of some cyber-bullying experiences compared to time spent on these sites
Egan et al. (2013)	To explore college students' views regarding the mental health status updates of their peers	Facebook	7 focus groups with 34 undergraduate students, 18-23 years old from one large public university (74% female)	Qualitative analysis based on grounded theory of small focus groups; as part of the focus group, participants also viewed hypothetical status updates that referenced being depressed or anxious to stimulate discussion	Although some participants viewed mental health status updates as "jokes" (not serious), many expressed genuine concern about these "calls for help"; willingness to act on concerns tended to parallel offline relationships (students felt comfortable approaching a close friend but not casual acquaintance or distant friend)

Moreno et al. (2009c)	To determine adolescents' interpretations of alcohol references displayed on SNS	SNS	32 individuals recruited from local recreation centers and clinics, 11-18 years old	Small focus groups of 3-6 participants, asked to discuss thoughts and interpretations of displayed alcohol references on adolescent SNS profiles that they have seen before, from which themes were identified	Regardless of whether displayed alcohol references represent actual use, adolescents typically interpret these references as representing actual use and acknowledge their potential influence on peer behavior
Nordfeldt et al. (2013)	To understand information-seeking behaviors, Internet use and social networking online in adolescents with type 1 diabetes (T1DM).	SNS	24 adolescent patients with T1DM recruited from a county hospital in southeast Sweden, 10-17 years old	Qualitative content analysis of audio and video-recorded focus groups (8 groups)	Participants expressed concerns about security and privacy of communicating online, the reliability of information presented, the frequency of updates in information presented and simplicity in design of a website
Yang & Brown (2013)	To examine associations between patterns of Facebook activity, motives for using Facebook, and late adolescents' social adjustment to the college environment	Facebook	193 college students (54% female) attending a Midwestern US university	Anonymous self-reported questionnaire regarding use of Facebook & social experiences in college	More frequent engagement in Facebook interactions was associated with better social adjustment & lower level of loneliness; Facebook status updating was related to poor social adjustment and higher levels of loneliness only for those who were less motivated to use Facebook to maintain existing relationships
Jelenchick et al. (2013)	To evaluate the association between social networking site (SNS) use and depression in older adolescents	SNS	190 college students (58% female), 18-23 years old	Initial online questionnaire, followed by ESM questionnaire administered over 7 days using SMS to personal cellphones, all regarding SNS use	Most participants reported low (53%) or average (39%) daily SNS use and only a minority (8%) reported high use; no evidence found supporting a relationship between SNS use & clinical depression

	using an experience sample method (ESM) approach				
van der Velden & El Emam (2013)	To understand how chronically ill teenage patients manage their privacy on social media sites	"Upopolis" (closed online SNS for young patients in Canada) & other common public SNS	20 hospital patients, 12-18 years old	Qualitative descriptive analysis of semi-structured interviews	The majority of patients do not use social media to connect with peer disease-sufferers, and use public SNS to maintain their existing social networks outside the hospital; patients experience high levels of social & psychological privacy as a result of their privacy-protective behavior and do not express concern about their informational privacy on Facebook
Vyas et al. (2012)	To examine SMS & social media utilization/behavior and how SMS & social media can be effectively used as part of public health interventions focused on decreasing sexual risk-taking among Latino youth	SNS, specifically asked about Facebook in qualitative interview phase	9th & 10th grade Latinos (57% female), 428 for questionnaire, 15 for interview	Mixed-methods: audio-assisted computer-based questionnaire followed by interviews regarding social media and SMS use	Social media & SMS use are pervasive among students, though Facebook may be slightly more feasible due to popularity & lack of access to mobile phones; program staff & students perceive these tools as credible and essential methods of communication; students expressed desire for 2-way messaging
Whiteley et al. (2011)	To investigate if frequency of cell phone & Internet use relates to psychological constructs (depression, life satisfaction, impulsivity) and to examine if frequency of	SNS	1518 African-Americans, 13-18 years old	Audio computer-assisted self-interview (ACASI)	Majority of participants used Myspace; association of increased cell phone frequency with sexual sensation seeking, impulsivity, & riskier peer sexual norms

	cell phone & Internet use relates to HIV/STI risk behaviors				
Veinot et al. (2011)	To examine how low-SES youth use information & communication technologies to socialize with others, and how this aligns with their communication about sexuality and HIV/STIs	SNS	94 individuals from a low-SES county, 14-24 years old	Descriptive analysis of 12 semi-structured focus groups	Young people frequently aired their concerns/fears of HIV/STI on SNS via spread of gossip/rumors, cautionary tales, etc.; many expressed hunger for credible information regarding sexual health, and expressed a desire to be supported/be a positive influence
van Rooij et al. (2010)	To explore the relationship between time spent on various Internet applications (including online gaming) & Compulsive Internet Use (CIU)	SNS, blogs	11094 secondary school students including 1421 longitudinal cohort, in the Netherlands (avg. age 14years old)	Paper questionnaire, including 14-item version of Compulsive Internet Use Survey	Strong link between online video gaming and CIU; changes in CIU over a year were shown to be most strongly related to changes in online gaming; strong association between CIU and downloading; SNS use has strongest association with CIU after gaming
Rice et al. (2010)	To examine the association between sexual health & internet/SNS use among homeless youths at risk for contracting HIV/AIDS	SNS	201 homeless adolescents, 13-24 years old	Computer-administered self-interview	84% of participants accessed the internet at least once a week; those who used the Internet/SNS to communicate with street peers were 5x more likely to engage in exchange sex, while those using to communicate with family was associated with a 68% reduction in odds of exchange sex

Rice et al. (2012)	To examine the acceptability of a youth-led, hybrid face-to-face & online social networking HIV prevention program for homeless youth	Myspace & Facebook, digital media projects done on YouTube, etc.	163 homeless youths, 13-25 years old	7 "Peer Leaders" recruited homeless youth (53) in the creation of digital media projects, and then recruited online youth (103) to participate in Myspace & Facebook communities where the projects were disseminated & discussed	The peer-led program was highly acceptable to homeless youth; social network analysis revealed that younger youth are disproportionately connected to same aged peers and females are disproportionately connected to other females; online SNS served as a great tool for participant retention as youth felt comfortable returning after missed sessions because their online presence meant they never 'left' the group; some youth leveraged their work in the program as an opportunity to reconnect with positive network ties by sharing online media they had created
Madan et al. (2011)	To determine whether adolescents with tracheostomies found Facebook to be a suitable environment in which to network with other patients with tracheostomies	Facebook	9 tracheostomy patients, 4 of which were actively involved on Facebook, 11-18 years old	9-item questionnaire regarding Facebook use	All 4 subjects shared the fact that they had a tracheostomy with the Facebook community; 3/4 subjects posted pictures of themselves with their trach sites visible; 3/4 subjects stated that they met and communicated with other Facebook members with tracheostomies
Woolford et al. (2013)	To explore patients' and parents' perspectives about developing a Facebook group as a component of a pediatric	Facebook Group	32 interviews with 11 patients with a BMI at or above 95th percentile for age and gender, 13-18 years old (78% female)	Qualitative analysis by constant comparative method of individual and small-group interviews with participants and their parents	The vast majority believed having a Facebook group would be a positive addition to the program and expressed few concerns; top potential benefits mentioned: allowing

	weight management program (MPOWER)		along with 21 selected parents (13 of whom were parents of adolescents; 8 were parents of younger patients)	who viewed a prototype MPOWER Facebook group developed using information gathered from previous quality improvement projects within the program	participants to stay connected, bond with each other, gain support for weight loss efforts, maintain motivation to make healthy choices; parents & adolescents emphasized importance of having a "secret" group and have appropriate monitoring and "rules"; nearly all parents reported privacy & security concerns related to social media but not to using Facebook for the program as they considered themselves Facebook "savvy"; parents also expressed interest in a similar group for themselves
Juvonen & Gross (2008)	To examine the overlap among targets of, and the similarities between, online and in-school bullying among Internet-using adolescents	SNS	1454 individuals (75% female), 12-17 years old	participants were recruited via a popular teen website (www.bolt.com) to complete an online questionnaire regarding experiences with "mean things" online, examined association between cyberbullying and social anxiety, the use of prevention tactics	Among heavy Internet users, cyberbullying is a common experience; the forms of online & in-school bullying are similar and experiences overlap; although some forms of electronic communication methods/devices are associated with elevated risk of cyberbullying, they are merely tools (not causes) of mean behavior; cyberbullying is associated with increased distress; youth rarely tell adults about their experiences and do not fully capitalize on the tools provided by communication technologies to prevent future

					incidents
Perren et al. (2010)	To investigate the associations between cyber versus traditional bullying and depressive symptoms in Swiss and Australian adolescents	SNS	2 samples: 374 students from Switzerland (grade 7-9), 1320 students from Australia (grade 8-10), mean age 13.8 years old	Questionnaire assessing penetration and victimization of traditional and cyber forms of bullying behavior in addition to scales assessing depressive symptoms	Victims of cyberbullying reported significantly higher levels of depressive symptoms even when controlling for the involvement in traditional bullying/victimization
Tucker et al. (2013)	To examine the reciprocal longitudinal associations between alcohol or other drug (AOD) -related media exposure and alcohol use among middle school students, and explore whether these associations differ by ethnicity or gender	SNS	2,321 students: 2,079 who completed first questionnaire and 1,787 who completed second questionnaire a year later, enrolled in grade 6-8 at 16 Southern California middle schools	Cross-lagged correlation analysis of data from two questionnaires to determine the temporal association between past month drinking and AOD-related media exposure	Greater AOD-related media exposure in 7th grade was significantly associated with a higher probability of alcohol use in 8th grade and alcohol use in 7th grade was marginally associated with greater AOD-related media exposure in 8th grade; experimenting with alcohol may encourage adolescents to seek out more AOD-related media content as a way to reinforce their emerging social identity as a drinker; impact on behavior was not different between different media types
Machold (2012)	To determine general patterns of Internet usage among Irish teenagers and to identify potential	SNS	460 Irish secondary school students, 11-16 years old	Questionnaire assessing general patterns and purpose of Internet usage, parental supervision and limitations, as	Two hours or less were spent online daily by 62% of participants, of whom 98% were unsupervised; 72% reported frequent usage of SNS, 95%

	hazards, including; bullying, inappropriate contact, overuse, addiction and invasion of users' privacy			well as hazards associated with SNS use	of whom were Facebook users; 10% males and 12% females experienced bullying online, while 27% reported inappropriate contact from others; concerning overuse and the risk of addiction, 33% felt they accessed SNS too often
Gowen et al. (2012)	Examine ways that young adults with mental illness 1) currently use social networking; and 2) how they would like to use a social networking site tailored for them	SNS	207 with and without mental illness, 18-24 years old	Online questionnaire assessing SNS habits and preferences to inform the creation of an SNS site for those with mental illnesses	Almost all (94%) participants with mental illnesses currently use SNS; those living with a mental illness are more likely than those not living with a mental illness to report engaging in various SNS activities that promote connectivity and making online friends, and are also more likely to report wanting resources on independent living skills and overcoming social isolation available through an SNS
Wolniczak et al. (2013)	To assess the potential association between Facebook dependence and poor sleep quality among university students in Peru	Facebook	418 undergraduate students from a Peruvian university, mean age 20.1 years old (77% women)	Questionnaire including Pittsburgh Sleep Quality Index (PSQI) and a questionnaire measuring Internet addiction adapted to assess Facebook dependence	"Facebook-dependent" participants had a 1.3 times higher prevalence of poor sleep quality compared to the non-dependent group; students with Facebook dependence also report more daytime dysfunction; about 8% displayed some degree of Facebook addiction

Campsi et al. (2012)	To examine perceptions of Facebook use and incidence of upper respiratory infections (URIs)	Facebook	88 individuals, 18-24 years old	Facebook-induced stress quantified via questions from a web-based questionnaire and weekly diary of URI symptoms	Social network size was significantly related to the rate of URI (the larger one's social network, the greater one's incidence of URI); most (85.7%) of respondents experienced some degree of Facebook-induced stress
Horgan & Sweeney (2012)	To determine university students' online habits and their use of the Internet for health information using a quantitative descriptive approach	SNS, including mention of Facebook, Bebo and Myspace	922 university students (62.4% female) from 1 Irish university, 18-24 years old	Use of internet for health information assessed via a 30-item questionnaire administered online over SurveyMonkey email link	66.1% if participants have used the Internet to search for general health information; information relating to sexual health (16.4%), as well as nutritional information, fitness, & weight loss, and sports-related injuries were most frequently searched; 34.6% reported searching for information on specific medical illnesses
Dowdell et al. (2011)	To gather information on students' and offenders' levels of engagement with SNS to report patterns as background information for health care professionals	SNS including Myspace, Facebook, & Second Life	404 middle school students, 2077 high school students; 1,284 students drawn from 5 traditional 4-year colleges (15-18 years old); 466 adult past sexual offenders	Questionnaires (Youth Internet Safety Survey, YISS; Youth Risk Behavior Surveillance Survey, YRBSS) assessing various characteristics of study participants' use of SNS	Students & offenders both frequent SNS; nearly 2/3 of Internet offenders initiated the topic of sex in their first chat session, more than 1/2 disguised their identity when online, preferred communicating with teen girls; high school students' experience with "sexting" differed according to sex; a small # of people are threatened/assaulted by people they meet online; use of Second Life is popular for both students & sex offenders

Landry et al. (2013)	To assess patterns of SMS & SNS use and their relationship to sexual behavior among a population of Latino adolescents	SNS	428 self-identifying Latino adolescents (58% female), 13-19 years old	Computer-based, audio-assisted questionnaire assessing social media use and sexual behavior	Participants who logged into their social media accounts at least once a day were significantly more likely to have ever had vaginal sex; increased frequency of logging into social media accounts was not significantly associated with contraceptive use
Li et al. (2013)	To assess the effectiveness of a fully automated, Web-based, social network electronic game on enhancing mental health knowledge and problem-solving skills of young people.	Facebook	73 students at a major university in Asia, 17-25 years old (58% female)	Online pre and post questionnaire: the first collected information on mental health literacy, whereas the second additionally collected modified questions for learning motivation	The gaming approach was effective in enhancing mental health literacy; intrinsic goal orientation was the primary factor in learning motivation, whereas test anxiety was successfully alleviated in the game setting; participants' self-efficacy for learning and performance positively influenced learning outcomes, whereas test anxiety negatively affected them
Struik et al. (2012)	To explore adolescent girls' perspectives on the use of SNS to deliver tobacco control (TC) messages targeting young women	SNS including Facebook, Myspace, Facebook Ads for recruitment	17 females, 16-19 years old	Qualitative analysis of semi-structured focus groups regarding current TC initiatives displayed in common media, thoughts on the use of SNS to deliver TC messages	Factors perceived as likely to influence TC message effectiveness on SNS: instant impact, message novelty, portrayal of women as self-assured, and gender stereotyping; suggested changes to current TC messages include the following themes: interaction, animation, and positive message framing; many TC messages directed towards girls are underpinned by assumption about gender

Black et al. (2013)	To examine the relationship between individual perception of and actual peer sexual risk behavior using online social networks	Facebook Pages	ethnically diverse group of 1,029 individuals from 162 virtual social networks, 16-25 years old	Online questionnaire through Zoomerang using validated constructs assessing a participant's own sexual behavior (protective & risky behavior) as well as perceptions of peer behavior	Participants significantly over-reported (estimated) risky behaviors of their peers and under-reported protective peer behaviors related to sex; participants perceive their peers are being more risky than they actually are
Litt & Stock (2011)	To examine impact of socially-based descriptive norms on willingness to drink alcohol, drinker prototype favorability, affective alcohol attitudes, and perceived vulnerability for alcohol-related consequences within the Prototype Willingness model	Facebook Profiles created for study purposes	189 adolescents from 5 private high schools, a swim team, and church youth group, 13-15 years old	Participants were assigned to 1 of 2 conditions (alcohol user vs. control), and viewed & rated a series of fabricated Facebook profiles, reactions of participants were assessed by questionnaire, data examined using multiple mediation analysis	Those who viewed Facebook profiles that portrayed alcohol use as normative among older peers reported greater willingness to use alcohol, more favorable images of alcohol users, more positive affective attitudes towards alcohol use, lower perceived vulnerability to the consequences of drinking alcohol, and greater perceived norms of alcohol use; normative influence of older peers had a significant impact on risk cognitions in adolescents
Young & Jordan (2013)	To test whether photos posted on social networking profiles may influence perceived social norms and thereby affect sexual health behavioral intentions	Public Facebook photos	Study 1: 49 private university students, 18-23 y.o; Study 2: 154 college-students, 18-25 y.o., recruited from Amazon's Mechanical Turk	Participants were randomly assigned to complete 1 of two online questionnaires: one which featured sexually suggestive photos and the other which featured non-sexually suggestive photos	Even a brief exposure to Facebook photos can convey information about peer norms, which in tern, may influence people's own sexual behavior intentions, owing to the importance of subjective norms in determining human behavior in general

Jones K et al. (2012)	To evaluate an evidence-based SNS intervention consisting of a Facebook site aimed at reducing the incidence of Chlamydia	Facebook Profile created for study purposes, "Caryn Forya"	70 individuals residing in a Midwestern US community, 18-24 years old	Questionnaire administered over Askpeople (internal health department survey software package), pre- and post intervention responses to STD diagnosis and testing were compared against county statistics	81% of subjects felt that the site influenced their decision to use sexual protection in future sexual experiences (23% increase in condom use following intervention); 74% felt that the intervention impacted their decision to reduce their # of sexual partners
Bull et al. (2012)	To determine whether STI prevention messages delivered via a Facebook page are efficacious in preventing increases in sexual risk behavior at 2 and 6 months	Facebook Pages	942 intervention (427 completed 6-month follow-up) and 636 control (377 completed 6-month follow-up) individuals, 16-25 years old	Participants randomized to intervention (Just/Us Facebook page) updated by youth facilitators with sexual health topics or control (18-24 News Facebook page) containing non-sexual health content; participants completed online questionnaire delivered via Zoomerang (online questionnaire software) to evaluate sexual health risk behaviors at 2 and 6 months	Intervention was effective in facilitating prevention of declines in condom use among high-risk individuals in the short term; effect size match or exceed those from meta-analysis of Internet interventions; recruitment strategy employing Facebook was successful in reaching youth of color/those living in areas with high STI/HIV prevalence; engagement with interventional content occurred almost exclusively on individuals' own pages; attrition among higher-risk youth is of concern
Livingston et al. (2013)	Evaluate the effectiveness of the In One Voice campaign for raising mental health awareness and improving attitudes of youth and young adults towards mental health	Mindcheck.ca	806 total individuals recruited through a market research company,13-25 years old, who were randomly divided into 2 equal samples	Online questionnaire pre- and post-campaign using two randomized groups to assess market penetration and attitudinal changes of the campaign	The proximal outcomes of the campaign to increase awareness and use of the website were achieved; the distal outcome of the campaign to improve attitudes towards mental health issues was not successfully achieved; the campaign improved mental health literacy

	issues				outcomes, but had limited effect on personal stigma and social distance
Gunasekaran et al. (2013)	To assess knowledge of cervical cancer/HPV among young women & investigate predictors of high knowledge	Facebook Ads for recruitment	278 females, 16-25 years old	Recruitment for a web-based questionnaire regarding HPV	Facebook ads resulted in efficient & cost-effective recruitment method; the geographic region, indigenous status, and SES of participants did not differ from Australian Bureau of Statistics distribution but women aged 16-17 and with lower education status were under-represented in study sample
Gamage et al. (2011)	To evaluate the effectiveness of advertising for "TESTme", an STI screening service to those in rural areas of Australia over an 11-month period	Facebook Page	28 rural young people, of which 1 was recruited via Facebook, 18-25 years old	Assessed direct cost of advertising through the different strategies, clients recruited & those who returned specimens, website visitors & page views	Advertising through paper-based methods, SMS, and SNS were not effective; the majority of those recruited accessed service through other HCP, or found it while looking for health services or health info online; Facebook page advertising incurred $1408
Lord et al. (2011)	To assess the feasibility of implementing a survey study about prescription medication misuse with college students on Facebook and identify the characteristics, motivations, beliefs, and attitudes associated with experimental	Facebook Ads	689 college students, 18-25 years old	Facebook advertisement to recruit for a 95-item questionnaire assessing nonmedical medication-related constructs; one open-ended question asking "What is your opinion about using prescription drugs that aren’t prescribe to you?” which was	Recruitment strategy was successful (average click-through rate of 0.02-0.04%); 27% of those who clicked on the ad completed consent, all despite lack of incentive for competing questionnaire; 61% of the sample reported having misused both prescription opioids and stimulants at some point in their lives

	vs. regular misuse of prescription opioid analgesic medications			qualitatively analyzed using a coding scheme	
Ahmed et al. (2013)	To examine potential barriers and facilitating factors for screening in young Victorian women using Facebook to recruit participants	Facebook Ads	278 women 16-25 years old from Victoria, Australia	Targeted recruitment using Facebook ads	Facebook ads yielded a total of 551 expressions from 7940 unique clicks: of these, 426 (77%) were contactable, all of whom were enrolled in the study; overall participation rate was 65% (n=278 out of 426)
Chu & Snider (2013)	Describe the effectiveness of using Facebook Ads for recruitment for a study evaluating the prevalence of PTSD in youth affected by violence	Facebook Ads	88 individuals who filled out surveys, 15-24 years old	Advertising performance data including cost of each click and impression and click-through-ratio	Total # of clicks & impressions were much higher on weekends; method was relatively inexpensive and required minimal personnel; Facebook's performance tracking allowed researchers to optimize their strategy more effectively
Kraaij & Garnefski (2012)	To examine relevant coping factors for the developments of psychological intervention programs for adolescents with a chronic medical condition	A Dutch SNS	101 adolescents self-identifying with a chronic medical condition (32% male) recruited from a Dutch SNS site and Internet forums along with 75 adolescents recruited from special schools for	Questionnaire assessing characteristics of one's chronic medical condition, negative life events, cognitive, behavioral, and goal adjustment coping, and depressive symptoms	Several cognitive and behavioral coping strategies and goal adjustment were found to be related to symptoms of depression. The cognitive coping strategies (self-blame, rumination and catastrophizing) had the strongest influence on depressive symptoms

			adolescents with a disability, 12-21 years old		
Ezell et al. (2013)	Analyze the results of four retention strategies used to reconnect with urban teens enrolled in a school-based RCT and overdue for a 12-month follow-up questionnaire.	Facebook	125 students overdue for 12-mth follow up out of 422 total enrolled students	Compared 4 retention strategies: re-mailing reminder letter, re-dialing non-working phone #, outreach on Facebook, obtaining assistance from school administration	42% of total attempts made to re-connect with the 125 students overdue for follow-up were successful; success rates for retention strategies: redialing non-working phone # on weekly basis (68%); obtaining assistance from school admin (57%); Facebook outreach (47%); mailed reminder letter (32%)
Huang et al. (2013)	To examine the use of SNSs among friends and the degree to which SNS activities relate to face-to-face peer influences and adolescent risk behaviors	Facebook, Myspace	1,563 10th-grade students from 5 high schools who completed questionnaires at 2 time points, mean age 15 years old	Questionnaire regarding tobacco/alcohol use, social media use, egocentric friend characteristics	Frequency of SNS use and # of closest friends on the same SNS were not significantly associated with risk behaviors, but exposure to friends' online photos of partying/drinking was significantly associated with both smoking and alcohol use; those with drinking friends had higher risk levels for drinking, but those without drinking friends were more likely to be affected by higher exposure to risky online photos; Myspace & Facebook has different demographics and differential effects on risk behaviors (higher levels of Myspace use were associated with alcohol use)
